# Four-component protein nanocages designed by programmed symmetry breaking

**DOI:** 10.1038/s41586-024-07814-1

**Published:** 2024-12-18

**Authors:** Sangmin Lee, Ryan D. Kibler, Green Ahn, Yang Hsia, Andrew J. Borst, Annika Philomin, Madison A. Kennedy, Buwei Huang, Barry Stoddard, David Baker

**Affiliations:** 1 Department of Biochemistry, University of Washington, Seattle, WA USA; 2 Institute for Protein Design, University of Washington, Seattle, WA USA; 3 Howard Hughes Medical Institute, University of Washington, Seattle, WA USA; 4 Department of Chemical Engineering, Pohang University of Science and Technology (POSTECH), Pohang, Republic of Korea; 5 Division of Basic Sciences, Fred Hutchinson Cancer Center, Seattle, WA USA

**Keywords:** Proteins, Protein design

## Abstract

Four, eight or twenty C3 symmetric protein trimers can be arranged with tetrahedral, octahedral or icosahedral point group symmetry to generate closed cage-like structures^1,2^. Viruses access more complex higher triangulation number icosahedral architectures by breaking perfect point group symmetry^3–9^, but nature appears not to have explored similar symmetry breaking for tetrahedral or octahedral symmetries. Here we describe a general design strategy for building higher triangulation number architectures starting from regular polyhedra through pseudosymmetrization of trimeric building blocks. Electron microscopy confirms the structures of T = 4 cages with 48 (tetrahedral), 96 (octahedral) and 240 (icosahedral) subunits, each with 4 distinct chains and 6 different protein–protein interfaces, and diameters of 33 nm, 43 nm and 75 nm, respectively. Higher triangulation number viruses possess very sophisticated functionalities; our general route to higher triangulation number nanocages should similarly enable a next generation of multiple antigen-displaying vaccine candidates^10,11^ and targeted delivery vehicles^12,13^.

## Abstract (web summary)

Using viral capsid architectures as template for design, higher triangulation number nanocages that require symmetry breaking offer potential advances in targeted delivery and antigen-displaying vaccines.

## Main

Natural and designed protein nanocages consist of one or multiple unique components arranged with tetrahedral, octahedral and icosahedral point group symmetry^1,3–7,14–16^. Although there are no other point group symmetries, in the icosahedral case, viruses access larger and more complex higher triangulation (T) number architectures by interspersing varying numbers of hexagons between 12 pentagonal substructures (pentons), which expand the structure without changing the curvature. Viruses achieve this symmetry breaking by placing at symmetrically non-equivalent positions either the same subunit in different conformations (quasisymmetry)^3–8^, or closely related but distinct subunits (pseudosymmetry)^9^. The accessing of higher T number icosahedral structures by such symmetry breaking is critical to the remarkable functionality of viruses, such as the ability to package and deliver large nucleic acid cargos. Similarly, the de novo design of higher T number protein assemblies could enable new approaches to nucleic acid delivery and, as the potency of nanoparticle immunogens can increase with increasing valency of display, could lead to more potent vaccines. However, although protein design has had considerable success in designing symmetric assemblies that assemble from identical interacting subunits^1,14–16^, the design of assemblies with multiple identical or nearly identical chains in non-symmetry equivalent positions is an outstanding challenge.

We set out to develop a systematic approach to design higher T-number protein assemblies that could generate not only icosahedral (I-sym) architectures but also tetrahedral (T-sym) and octahedral (O-sym) higher T-number nanostructures, which, to our knowledge, have not been found in nature. Of the two routes to breaking symmetry described above, we reasoned that using closely related but distinct subunits (pseudosymmetry) would have the advantage over quasisymmetry as it avoids the complexity of designing single subunits with multiple distinct states and sets of interactions. We reasoned further that a design strategy that started with creating T = 1 architectures from homotrimers, and then extended these to T = 4 by inserting pseudosymmetric heterotrimers, would be more robust than directly designing for higher T-number cages, as subsets of the cage forming interfaces could be experimentally validated independently.

**Fig. 1:**
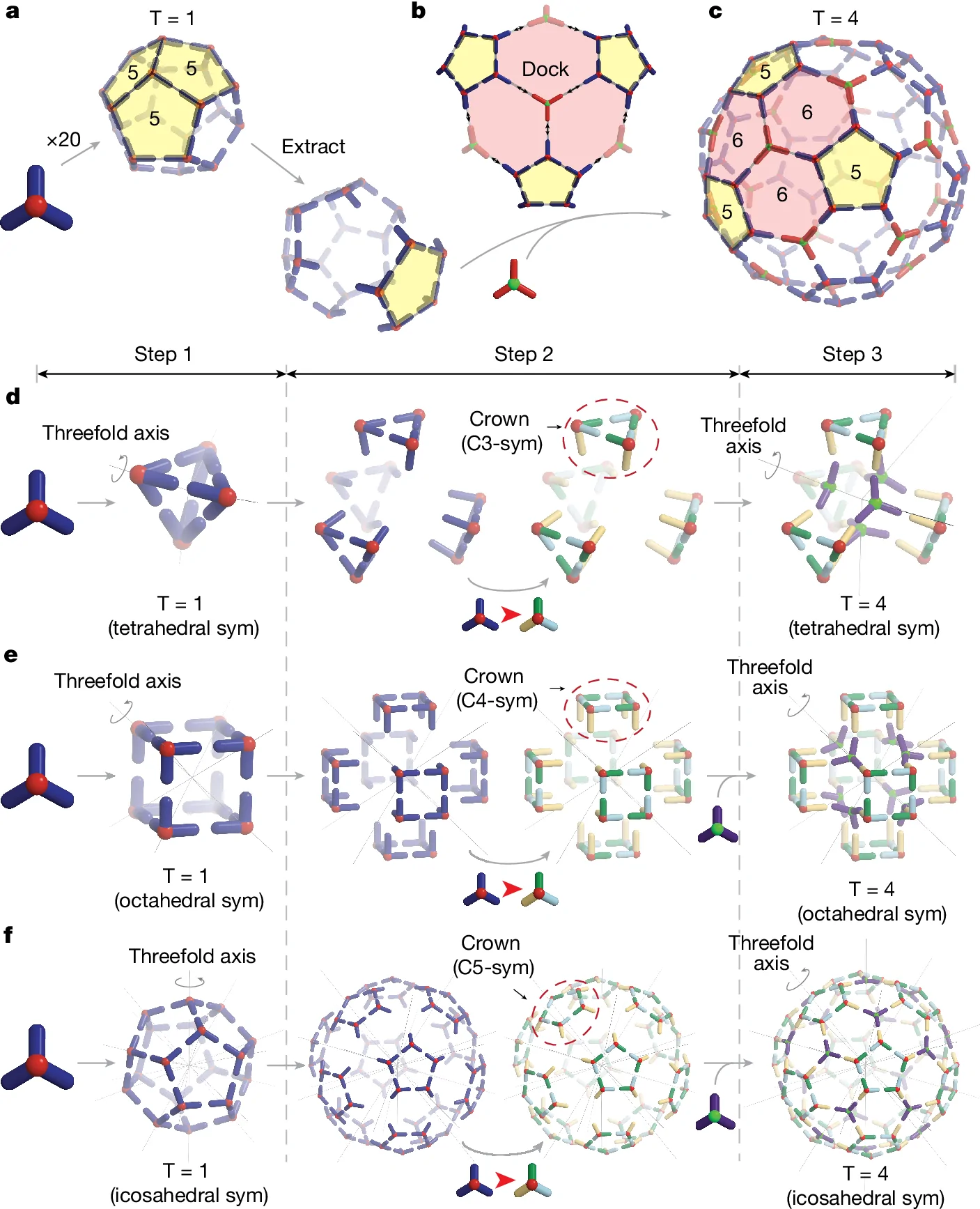
Overview of the design strategy. **a**–**c**, General design route to T = 4 icosahedral cages using substructures extracted from a T = 1 cage. Twelve pentagonal substructures (pentons) from a T = 1 cage (**a**) are docked with 20 homotrimers to form a closed cage structure (**b**), which creates hexagonal local structures (shaded red) placed between pentons (shaded yellow; **c**). **d**–**f**, Schematic of the route to T = 4 tetrahedral (**d**), octahedral (**e**) and icosahedral (**f**) cages. Step 1: T = 1 cages are designed starting from C3 symmetric trimeric building blocks. Step 2: the trimers constituting each face of the T = 1 cages are displaced away from the origin along the symmetry axis orthogonal to the face (left) and replaced by ABC-type pseudosymmetric heterotrimers to produce crowns in which one of the three components (yellow) is free to be designed to dock to other building blocks (right). Step 3: the crowns are docked with a new set of homotrimers aligned along the threefold symmetry axis of each architecture, which produces T = 4 cages.

Our design strategy is illustrated in Fig. 1a for the icosahedral case. A T = 1 cage with icosahedral point group symmetry can be built from 20 homotrimers aligned along the icosahedral threefold symmetry axes (Fig. 1a). The centres of the homotrimers can be visualized as the vertices of 12 pentagonal faces that form the sides of a dodecahedron (Fig. 1a). Higher T-number cages conserve the number of pentagons (pentons), which are always aligned along the 12 icosahedral fivefold symmetry axes. T = 4 cages with hexagons bridging the 12 pentons could be generated if it were possible to extract pentons from the T = 1 cages and insert homotrimers along the icosahedral threefold symmetry axes connecting three pentons (Fig. 1b,c). Extracting pentons from T = 1 cages requires that one of the three interfaces joining each trimer with its nearest neighbours be eliminated. This can be accomplished by substituting the homotrimers with geometrically identical (that is, pseudosymmetric) heterotrimers composed of three distinct chains with different amino acid sequences. This symmetry breaking allows the interface facing outwards from the penton to be specifically eliminated, isolating the penton as a free-standing structure that can be used to build larger structures.

We reasoned that this approach could be used to design T = 4 tetrahedral, octahedral and icosahedral nanostructures using pseudosymmetric heterotrimers to precisely programme the six distinct interfaces in such structures. We adopted the three-step hierarchical approach outlined in Fig. 1d–f. We first designed T = 1 cages by arranging C3 symmetric homotrimers along the threefold symmetry axis of each architecture^1,2^ (Fig. 1d–f, step 1). Following experimental validation, we next extracted cyclic ‘crown’-like substructures from these cages (pentons in the icosahedral case) by replacing the symmetric homotrimers with ABC-type heterotrimers that lack the cage-forming trimer–trimer interface on one of the subunits, and redesigning the crown-forming trimer–trimer interface to be heterodimeric (Fig. 1d–f, step 2). Following experimental validation, these crown-like structures are arranged along one of the threefold symmetry axes of T-sym, the fourfold symmetry axis of O-sym or the fivefold symmetry axis of I-sym architectures, and docked and designed to interact with an additional symmetric homotrimer placed on the remaining threefold axis (Fig. 1d–f, step 3). This generates four component T = 4 cages for each cage symmetry, with a hexameric motif between edges of the crowns (*h* = 2 and *k* = 0 in the Caspar and Klug nomenclature^8^).

## Design of pseudosymmetric heterotrimers

**Fig. 2:**
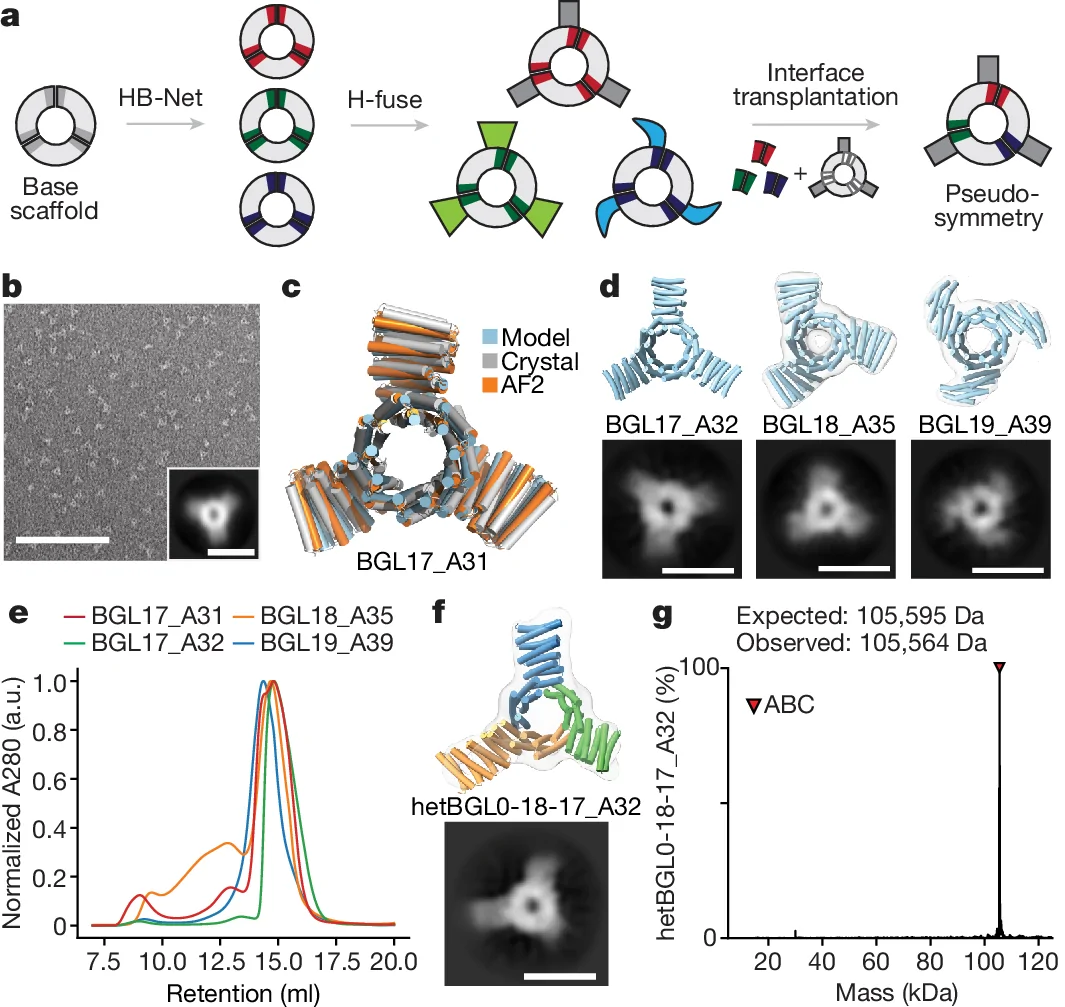
Design of pseudosymmetric ABC heterotrimers with extensible arms. **a**, Schematic of the design strategy for homotrimers with arms and pseudosymmetric heterotrimers. **b**, nsEM micrograph of the BGL17_A31 homotrimer and a 2D class average (inset) along a threefold symmetry axis. **c**, Structure of BGL17_A31 solved by X-ray crystallography (white) compared with its design model (light blue) and AF2 prediction (orange). **d**, Superpositions of the 3D-reconstructed nsEM map (transparent cloud) to the structure model (blue) of BGL17_A32 (no map; top left), BGL18_A35 (top middle) and BGL19_A39 (top right). 2D class averages along threefold symmetry axis are also shown (bottom). **e**, SEC results of homotrimers. **f**, ABC-type pseudosymmetric heterotrimer (hetBGL0-18-17_A32) with arms of different lengths extending from each component. Top, the 3D-reconstructed nsEM map (transparent cloud) superimposed on the design model (colours indicate the three different chains). Bottom, a 2D class average along the pseudo-threefold symmetry axis. **g**, nMS analysis of hetBGL0-18-17_A32; only the heterotrimeric ABC species is observed. Scale bars, 100 nm (**b**) and 10 nm (**b** (inset),**d**,**f**). See Extended Data Table 1 for the amino acid sequences of the oligomers.

Our design strategy requires homotrimeric building blocks that can be replaced by pseudosymmetric heterotrimeric blocks with distinct intra-trimer interfaces but identical outwards-facing interfaces that mediate nanocage assembly. We chose to utilize a previously designed family of ring-shaped homotrimers and heterotrimers^17^, which have the same structure but different amino acid sequences at the protomer–protomer interfaces. The heterotrimer designs contain all three chains in equal stoichiometries, and both homotrimer and heterotrimer designs assemble into symmetric cyclic rings very close to the computational design models^17^. To generate a wider diversity of geometries to enable docking of the rings into closed architectures through helix–helix interfaces (Fig. 2a), we built on previous work^17–19^ and rigidly fused various helical repeat protein extensions onto the termini^19^ (referred to as ‘arms’ below), yielding homotrimers of approximately 10 nm in diameter (Fig. 2b–e, Supplementary Table 2, Supplementary Fig. 1 and Supplementary information 1). Of the designs, 37 of 39 were soluble following expression in *Escherichia coli* and purification from immobilized metal affinity chromatography, 33 of 39 had size-exclusion chromatography (SEC) retention volumes consistent with the design models and the SEC profiles were monodisperse for 22 of the designs (Supplementary Figs. 2 and 3). The homotrimeric state was confirmed by native mass spectrometry (nMS) for 27 of 39 designs (Supplementary Figs. 4–6), and small-angle X-ray scattering profiles were closely consistent with profiles computed from the design models for 19 (Supplementary Figs. 7 and 8). Negative-stain electron microscopy (nsEM) characterization of the overall shapes of the designs was again consistent with the design models, with curved, straight, wide and narrow arm arrangements evident in 2D class averages and 3D reconstructions (Fig. 2b,d and Supplementary Fig. 9). We obtained a crystal structure of one of the designs, BGL17_A31 (see Supplementary information 1.3 for protein naming), which was very close to the design model and AlphaFold2 (AF2) prediction (Fig. 2c and Extended Data Table 2).

To design pseudosymmetric heterotrimers, we used an interface transplantation approach (Supplementary Figs. 10 and 11), preserving the overall structural C3 symmetry (Supplementary information 2). A homotrimer was selected as the host scaffold and three different protomer–protomer interfaces from different homo-oligomers (guests) were transplanted onto the subunit–subunit interfaces (Fig. 2a and Supplementary Table 1), conserving the residues at the arm junction. We first checked the compatibility between a host and a guest in the homo-oligomer context by symmetrically transplanting the guest interface into the host. We selected highly expressed and homogenous homotrimers for hybridization: seven with arms (BGL0_A10, BGL0_A11, BGL17_A31, BGL17_A32, BGL18_A35, BGL19_A38 and BGL19_A39) as host scaffolds and four without arms (BGL0, BGL17, BGL18 and BGL19)^17^ as guest interfaces. We experimentally characterized 18 of the 28 combinations (Supplementary Table 3); 14 of 18 had SEC peaks at the correct oligomeric size (Supplementary Fig. 12) and 11 of 18 had strong nMS signals at the correct masses (Supplementary Fig. 13). The BGL17_A32 host backbone was found to be compatible with multiple guest interfaces; we constructed heterotrimers by splicing interfaces from different guests together in different combinations^17^ (Supplementary Table 4). To enable assignment of chain type by electron microscopy, we varied the number of repeat units on the arms protruding from each chain (−1 repeat for the A component and +1 to the C component). Three of five heterotrimers (hetBGL0-17-19_A32, hetBGL0-18-17_A32 and hetBGL0-19-17_A32; see Supplementary information 2 for protein naming) formed ABC-type heterotrimers as shown by SEC, SDS–PAGE and nMS (Fig. 2f and Supplementary Figs. 14–17). For two of these (hetBGL0-18-17_A32 and hetBGL0-19-17_A32), the expected differences in arm lengths were clearly evident in 2D nsEM averages and 3D reconstructions (Fig. 2f and Supplementary Fig. 18). This approach to creating pseudosymmetric hetero-oligomers should be readily applicable to symmetric protein oligomers quite generally, if the amino acid sequence of the protomer–protomer interface can be diversified.

## Cage design

**Fig. 3:**
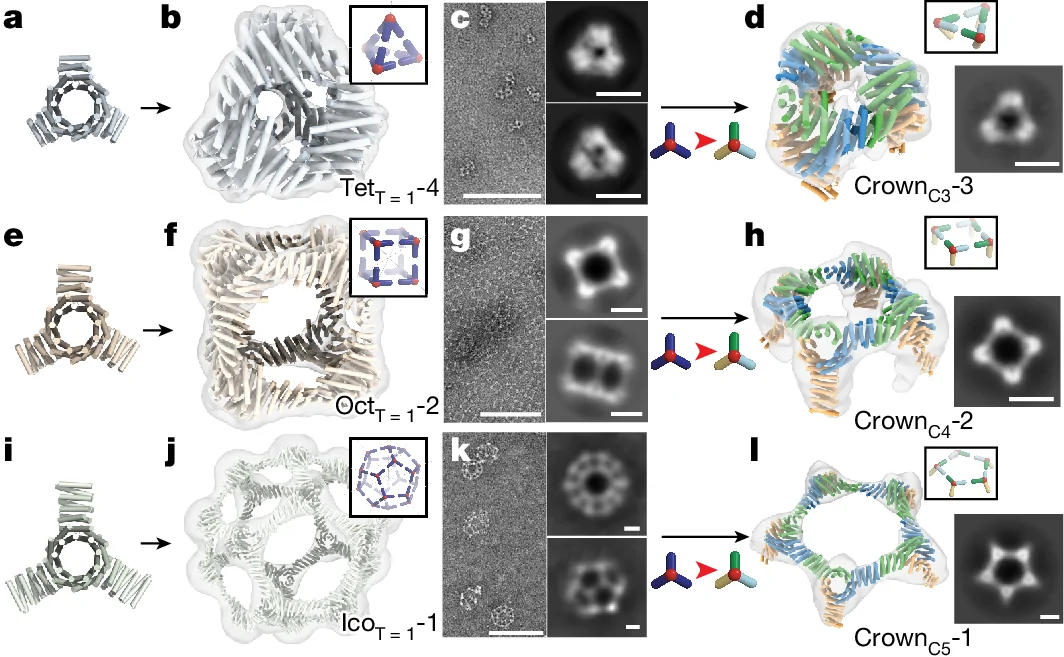
Extraction of homotrimer cycles (crowns) from T = 1 cages by pseudosymmetrization. **a**–**c**,**e**–**g**,**i**–**k**, BGL17_A32 with 1.5 (**a**), 2.5 (**e**) and 3.5 (**i**) repeat unit arms docked into tetrahedral (Tet_T = 1_-4; **b**,**c**), octahedral (Oct_T = 1_-2; **f**,**g**) and icosahedral (Ico_T = 1_-1; **j**,**k**) T = 1 cages. Superpositions of the 3D-reconstructed nsEM map (transparent cloud) on the cage design model (colours) are shown (**b**,**f**,**j**). nsEM micrographs (left) and characteristic 2D class averages (right) of the cages are also displayed (**c**,**g**,**k**). **d**,**h**,**l**, C3 (crown_C3_-3), C4 (crown_C4_-2) and C5 (crown_C5_-1) crowns made from pseudosymmetric heterotrimers. Left, superpositions of the 3D-reconstructed nsEM map on the crown design model (ch_A (green), ch_B (blue) and ch_C (orange)). Right, 2D class averages along threefold, fourfold and fivefold symmetry axes. The diameters of the crowns are 11 nm (C3), 20 nm (C4) and 35 nm (C5). Scale bars, 100 nm (**c**,**g**,**k** (left)) and 10 nm (**c**,**g**,**k** (right),**d**,**h**,**l**). See Extended Data Tables 3 and 4 for the amino acid sequences of the T = 1 cages and crowns.

**Extended Data Fig. 1:**
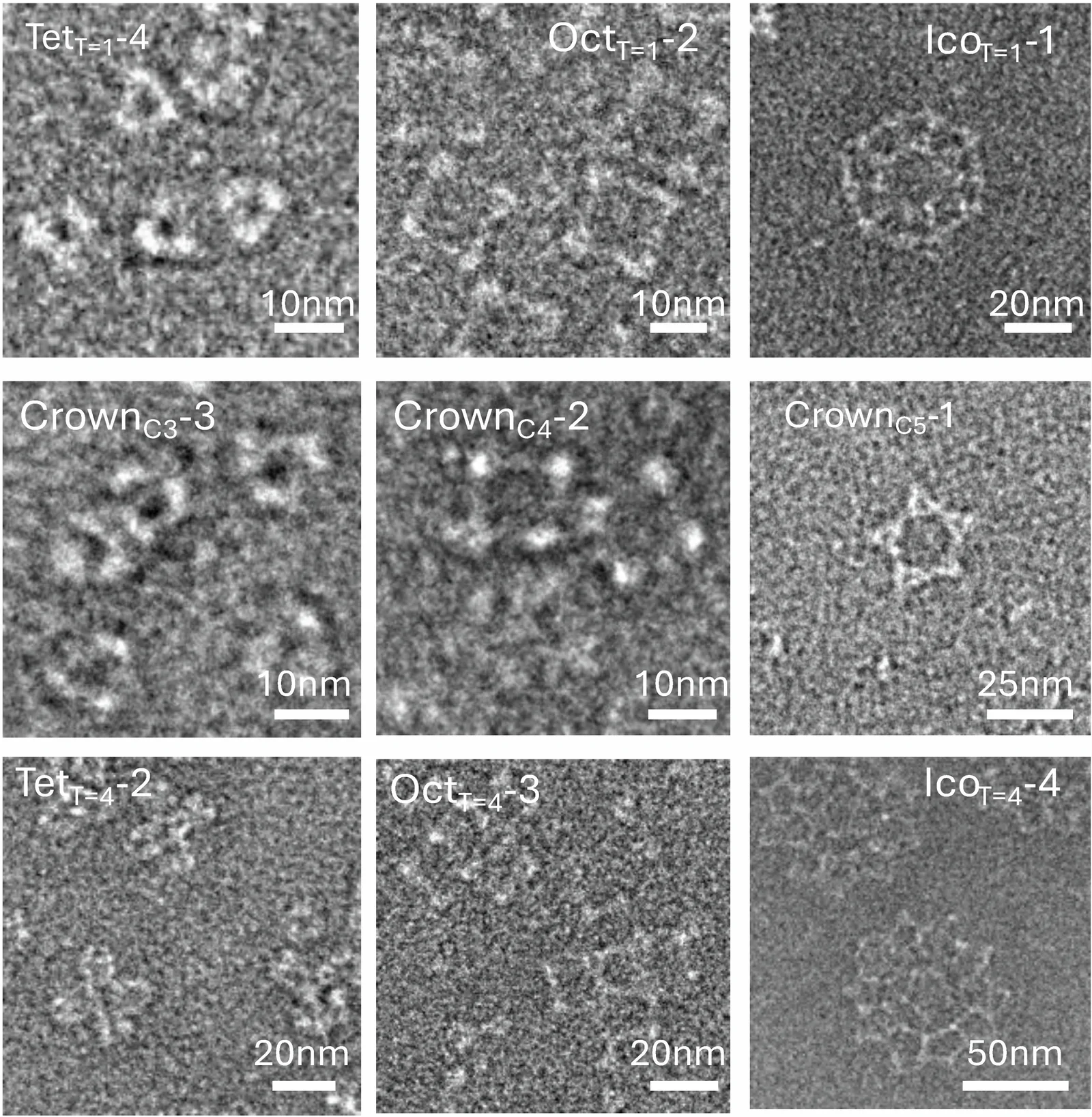
Zoomed-in view of nsEM images. T = 1 cages (top), crowns (middle), T = 4 cages (bottom).

We generated base T = 1 tetrahedral, octahedral and icosahedral cages from the BGL17_A32 homotrimer using RPXdock^20,21^ to sample rotational and translational displacements of the trimer C3 axis along the threefold cage axes (Supplementary information 3). We found that for the different symmetries, different numbers of repeat units of arms (1.5, 2.5 and 3.5 repeat units for tetrahedral, octahedral and icosahedral cages, respectively) were optimal for docking of the trimers with good shape complementarity (Fig. 3a,b,e,f,i,j and see Supplementary information 3.1). The newly generated cage interfaces were designed using ProteinMPNN^22^, and designs for which the AF2 prediction of the arm–arm interface was less than 2.0 Å root-mean-square deviation (RMSD) from the design model were selected for experimental characterization; these comprise seven tetrahedral cages (Tet_T = 1_-1 to Tet_T = 1_-7; Supplementary Table 5) with 12 subunits and diameters of approximately 13 nm, 8 octahedral cages (Oct_T = 1_-1 to Oct_T = 1_-8; Supplementary Table 6) with 24 subunits and diameters of approximately 20 nm, and 4 icosahedral cages (Ico_T = 1_-1 to Ico_T = 1_-4; Supplementary Table 7) with 60 subunits and diameters of approximately 40 nm. All of the tetrahedral designs had peaks at the expected retention volume (Supplementary Fig. 20) on SEC, and four (Tet_T = 1_-1, Tet_T = 1_-2, Tet_T = 1_-4 and Tet_T = 1_-6) were structurally homogeneous by nsEM (Extended Data Fig. 1 and Supplementary Fig. 21), with 2D class averages and 3D-reconstructed nsEM maps (Fig. 3c and Supplementary Fig. 21) matching the design models (Fig. 3b,c). Seven of the octahedral cages had single peaks on SEC, and two (Oct_T = 1_-2 and Oct_T = 1_-4) showed homogenous structures matching the design models by nsEM (Fig. 3f,g and Supplementary Figs. 22 and 23). One of the icosahedral cages, Ico_T = 1_-1, had a single peak on SEC and was close to the design model by nsEM (Fig. 3j,k), although imperfectly formed cages were also observed (Supplementary Fig. 24).

We next extracted C3, C4 and C5 symmetric cyclic oligomers (which we refer to as crowns because of their shape) from the structurally confirmed T = 1 cages by substituting in the structurally identical pseudosymmetric hetBGL0-18-17_A32 heterotrimer in place of the BGL17_A32 homotrimer (Fig. 1, third and fourth columns). The chain A (ch_A) and ch_B interfaces were redesigned using ProteinMPNN to interact at the crown trimer–trimer interface; this was necessary to avoid a potential off-target structure possible with the original C2 interface (Supplementary Fig. 25). The surface of the arm of ch_C, which points outwards from the crown, was redesigned to be entirely polar. This isolates the crowns as free-standing building blocks that can be used to build T = 4 cages in the next step. We selected designs for which AF2 predicted the ch_A–ch_B interface with RMSD < 2 Å and did not predict the ch_A–ch_A or ch_B–ch_B homodimer interfaces to form (Supplementary Fig. 19). We obtained genes encoding 19 sets of crowns that passed these filters (crown_C3_-1 to crown_C3_-5 for C3 crowns, crown_C4_-1 to crown_C4_-7 for C4 crowns and crown_C5_-1 to crown_C5_-7 for C5 crowns; Supplementary Tables 8–10), and the three chains for each crown were expressed separately in independent *E. coli* cultures. Following expression, the amount of each protein was estimated by SDS–PAGE gel densitometry, and appropriate amounts of culture were combined to achieve mixtures with stoichiometric amounts of the three chains, which were co-lysed and co-purified. By SEC, four of five C3, five of seven C4 and two of seven C5 crowns had peaks at the expected elution volumes (Supplementary Figs. 26–31) containing three distinct bands by SDS–PAGE (Supplementary Fig. 32), indicating that the complexes were heterotrimers. nsEM 2D class averages and 3D-reconstructed nsEM maps matched well with the crown design models (Fig. 3d,h,l and Extended Data Fig. 1). Thus, symmetric substructures can be extracted from larger symmetric assemblies by substituting homotrimers with pseudosymmetric heterotrimers.

In the final step of our hierarchical design approach, we designed T = 4 cages by combining the experimentally confirmed crowns with the BGL17_A32 homotrimer (Fig. 1, last column, and Supplementary information 3). The C3, C4 and C5 crowns were aligned with the threefold, fourfold and fivefold axes of tetrahedral, octahedral and icosahedral architectures, and BGL17_A32 was aligned to the remaining threefold axis. This generated assemblies in which the heterotrimer arms pointing outwards from the crowns (ch_C of heterotrimer) interact with the arms of the homotrimer (ch_ho). To find optimal docking interfaces, we used RPXdock, sampling the lengths of the interacting arms and the rotations and translations along the common axis, and designed sequences using proteinMPNN for the highest RPX scoring models for each symmetry. Designs were filtered based on the formation of the designed interface, and lack of formation of the self interfaces, in AF2 predictions (Supplementary Fig. 19). Very few initial designs for the octahedral architecture did not form predicted self interfaces; thus, to decrease the probability of self-interaction, we performed explicit negative design using proteinMPNN^22^ against the predicted self interfaces. We experimentally tested 14 sets of T = 4 cage designs (Tet_T = 4_-1 to Tet_T = 4_-5, Oct_T = 4_-1 to Oct_T=4_-4 and Ico_T = 4_-1 to Ico_T = 4_-5; Supplementary Tables 11–13) that passed the AF2 filters. The four components were expressed independently in different *E. coli*. cultures, mixed with 1:1:1:1 stoichiometry and co-lysed. The lysed samples were purified using immobilized metal affinity chromatography and SEC, and the cage structures were characterized by nsEM (Extended Data Fig. 1 and Supplementary Figs. 35–47). As described in the following paragraphs, the major species in each case was the designed T = 4 structure; we also observed minor species of smaller off-target T = 1-like cages (Supplementary Figs. 37, 42 and 45).

**Fig. 4:**
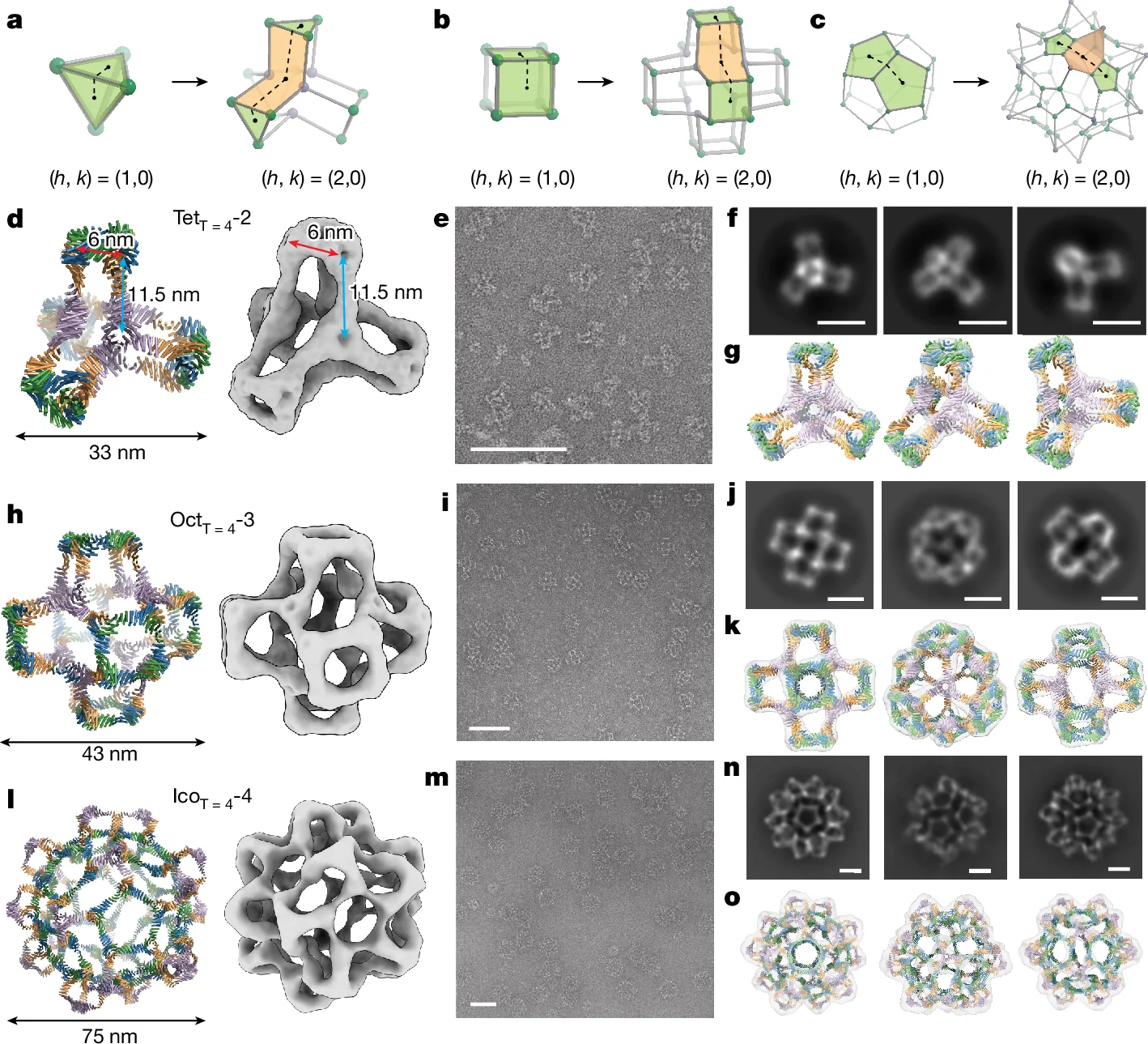
nsEM characterization of designed T = 4 tetrahedral, octahedral and icosahedral protein cages. **a**–**c**, Ball-and-stick models of T = 1 (left) and T = 4 (right) cages for each symmetry, defined by the Caspar and Klug nomenclature^8^. **d**–**o**, Four component T = 4 tetrahedral cage with 48 subunits (**d**–**g**), octahedral cage with 96 subunits (**h**–**k**) and icosahedral cage with 240 subunits (**l**–**o**). Design models of the T = 4 cages (left) and a 3D-reconstructed nsEM map (right) are shown (**d**,**h**,**l**). In the design models, each chain is shown in a distinct colour (ch_A (green), ch_B (blue), ch_C (orange) and ch_ho (purple)). nsEM micrographs (**e**,**i**,**m**), characteristic 2D class averages of nsEM (**f**,**j**,**n**) and superpositions of the 3D-reconstructed nsEM map (grey density) on the cage design model (colours; **g**,**k**,**o**) are also displayed. Scale bars, 100 nm (**e**,**i**,**m**) and 20 nm (**f**,**j**,**n**). See Extended Data Table 5 for the amino acid sequences of the T = 4 cages.

The T = 4 tetrahedral cage (Tet_T = 4_-2) has a tetrapod shape (diameter of 33 nm) with the four C3 crowns pointing outwards, and the homotrimers bridging the crowns closer to the centre of the cage and facing inwards (Fig. 4a,d,g and Supplementary Fig. 36). The homotrimer–heterotrimer distance (11.5 nm) is almost twice the heterotrimer–heterotrimer distance (6.0 nm) due to the arm length difference between components (1.5 repeat units for ch_A and ch_B, and 3 repeat units for ch_C and ch_ho), and the interior volume is a tetrahedral channel 6.0 nm in width (Fig. 4d). Overall, the structure maps to a T = 4 Goldberg polyhedra with tetrahedral symmetry^23^, in which the hexagonal motifs between triangles are highly elongated (Fig. 4a). These structural features are evident in the nsEM map (Fig. 4d), micrographs (Fig. 4e) and 2D average classes (Fig. 4f), and the design model is closely consistent with the reconstructed nsEM map (Fig. 4g). The design model could be readily relaxed to fit the nsEM 3D map with all four components clearly within density (Supplementary Fig. 36); overall, the relaxed model matches well with the design model, with the exception of a slight twist of the overall structure resulting from curvature in the arm near the homotrimer–heterotrimer interface (Fig. 4d and Supplementary Fig. 36).

**Fig. 5:**
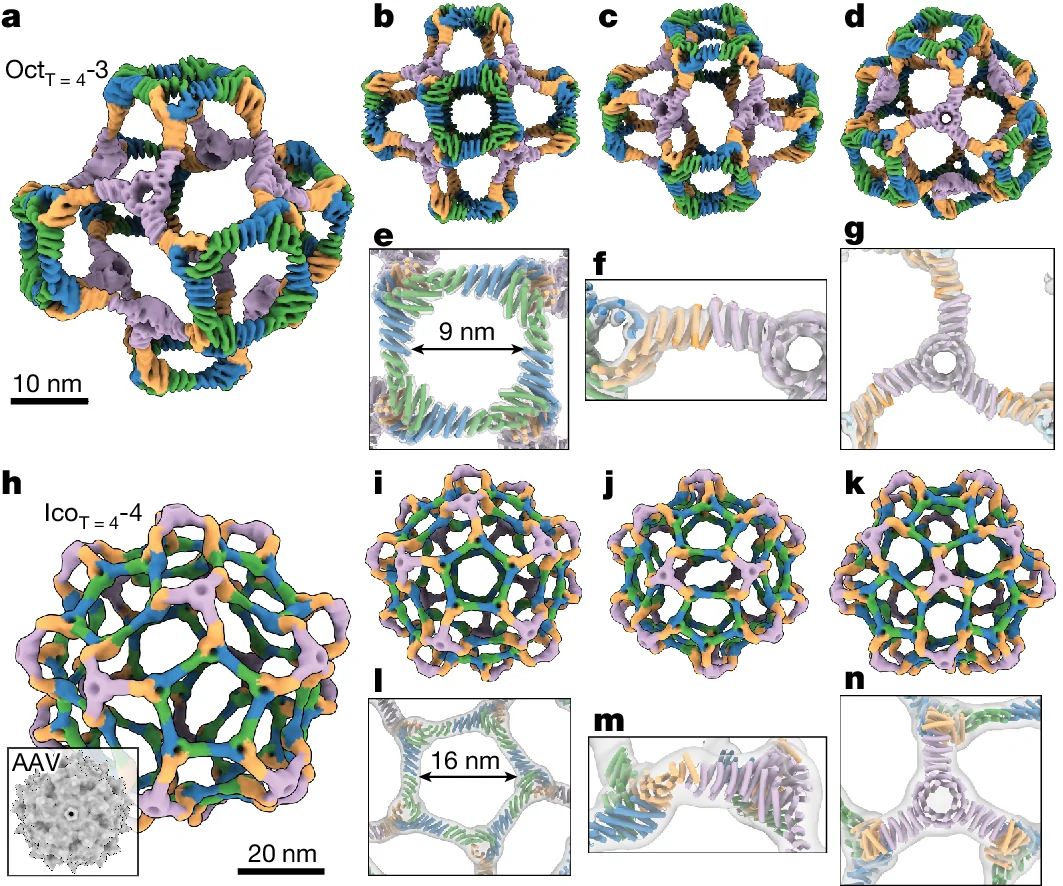
Cryo-EM characterization of T = 4 octahedral and icosahedral protein cages. **a**–**d**, 3D cryo-EM map of Oct_T = 4_-3 from different views. **e**–**g**, Overlay between the cryo-EM map (grey transparent) and the design model relaxed into the map (colours) for each substructure: C4 crown (**e**), the homotrimer–heterotrimer interface (**f**) and the homotrimer (**g**). **h**–**k**, 3D cryo-EM map of Ico_T = 4_-4 from different views. The inset (**h**) is an adeno-associated virus (AAV) capsid shown at the same scale for size comparison. **l**–**n**, Overlay between the cryo-EM map (continuous density) and the relaxed design model (colours) for each substructure: C5 crown (**l**), the homotrimer–heterotrimer interface (**m**) and the homotrimer (**n**). Both the cryo-EM map and the protein model are coloured by chain (ch_A (green), ch_B (blue), ch_C (orange) and ch_ho (purple)). See Extended Data Table 7 for cryo-EM data collection information.

The T = 4 octahedral cage (Oct_T = 4_-3) has a 3D cross shape structure (diameter of 43 nm) with the original cubic shape of the T = 1 structure repeated six times and shifted away from the origin to positive and negative values of *x*, *y* and *z* along the three fourfold symmetry axes (Fig. 4b,h). Six C4 crowns form the outward faces of the structure along the *x*, *y* and *z* axes, which are connected by eight homotrimers placed in a cubic arrangement closer to the centre of the cage; as for the tetrahedral cage, the homotrimers and heterotrimers face in opposite directions (Supplementary Fig. 40). The overall architecture is that of a T = 4 Goldberg polyhedra with octahedral symmetry^23^, with an elongated hexagon bridging the square faces (Fig. 4b) and a 10-nm cavity in the centre. Homogeneous populations of cages are observed in nsEM micrographs (Fig. 4i), and 2D class averages along the twofold, threefold and fourfold symmetry axes are closely consistent with the design model (Fig. 4j). The nsEM 3D reconstruction is very close to the design model (Fig. 4k), but a slightly curved connection between crowns and homotrimers leading to a slight twist of the overall structure (Supplementary Fig. 40). To characterize the structure of Oct_T = 4_-3 at higher resolution, we collected cryo-electron microscopy (cryo-EM) data and generated a 3D reconstruction, which, following refinement, resulted in a 3D cryo-EM map with 6.87 Å resolution with clear secondary structure features (Fig. 5a–d and Supplementary Fig. 51). Following relaxation via molecular dynamics^24^, the design model fits well into the cryo-EM map (Fig. 5e–g), with the crown and homotrimer substructures and the individual chains clearly defined (Supplementary Fig. 41). Around the fourfold symmetry axes, ch_A and ch_B of the hetBGL0-18-17_A32 heterotrimers (Fig. 5b,e, green and blue) form square motifs; the five helices in each arm in both chains are clearly evident in the cryo-EM map (Fig. 5e). BGL17_A32 homotrimers are placed along the threefold symmetry axes (Fig. 5d); the arm of each subunit has six helices and forms an interface with ch_C of the heterotrimer at the end (Fig. 5f,g, purple and orange). The slight twist of the C4 crown substructures relative to the design model arises from shifts at the homotrimer–heterotrimer interface (Supplementary Fig. 41).

The T = 4 icosahedral cage (Ico_T = 4_-4) consists of 12 C5 crowns (pentons) connected by 20 outwards-facing homotrimers (Fig. 4c,l). Largely homogeneous 75-nm-sized cages were identified by nsEM and dynamic light scattering (DLS; Fig. 4m and Supplementary Fig. 44). SDS–PAGE showed clear bands corresponding to each component, suggesting that all four chains are present (Supplementary Fig. 43). The 2D class averages (Fig. 4n) and 3D nsEM reconstructions (Fig. 4l,o) have the overall designed shape but the orientations of the C5 crowns, and homotrimers appeared inverted from the design model (Supplementary Figs. 34 and 47). We collected cryo-EM images, and 3D reconstruction and refinement of the cryo-EM data yielded a 3D cryo-EM map with 13.15 Å resolution (Fig. 5h–k and Supplementary Fig. 51), in which the holes at the centre of trimers and the orientations of trimers are clearly identified. The design model with inverted components fits well into the cryo-EM density following relaxation (Fig. 5l–n and Supplementary Fig. 48). The overall structure has the architecture of a T = 4 Goldberg polyhedron with icosahedral symmetry^23^, with boat-type hexagonal motifs placed between pentagons (Fig. 4c). Surrounding the fivefold symmetry axes are pentons formed from hetBGL0-18-17_A32 heterotrimers (Fig. 5h,i, green, blue and orange). The pentons are bridged by BGL17_A32 homotrimers, which form tripod-like protrusions on the threefold symmetry axis (Fig. 5h,k, purple). On the twofold axes are boat-type distorted hexagons with two homotrimers and four heterotrimers on the vertices; two of the edges are formed by interacting heterotrimer subunits, and four edges are formed by interacting homotrimer and heterotrimer subunits (Fig. 5j). The outer diameter of the Ico_T = 4_-4 cage is about three times larger than that of the adeno-associated virus capsid (Fig. 5h), and the inner diameter of the empty pore at the centre of the Ico_T = 4_-4 cage is approximately 50 nm (volume of approximately 6.55 × 10^4^ nm^3^), which can be used to package diverse cargos such as nucleic acids and enzymes.

**Fig. 6:**
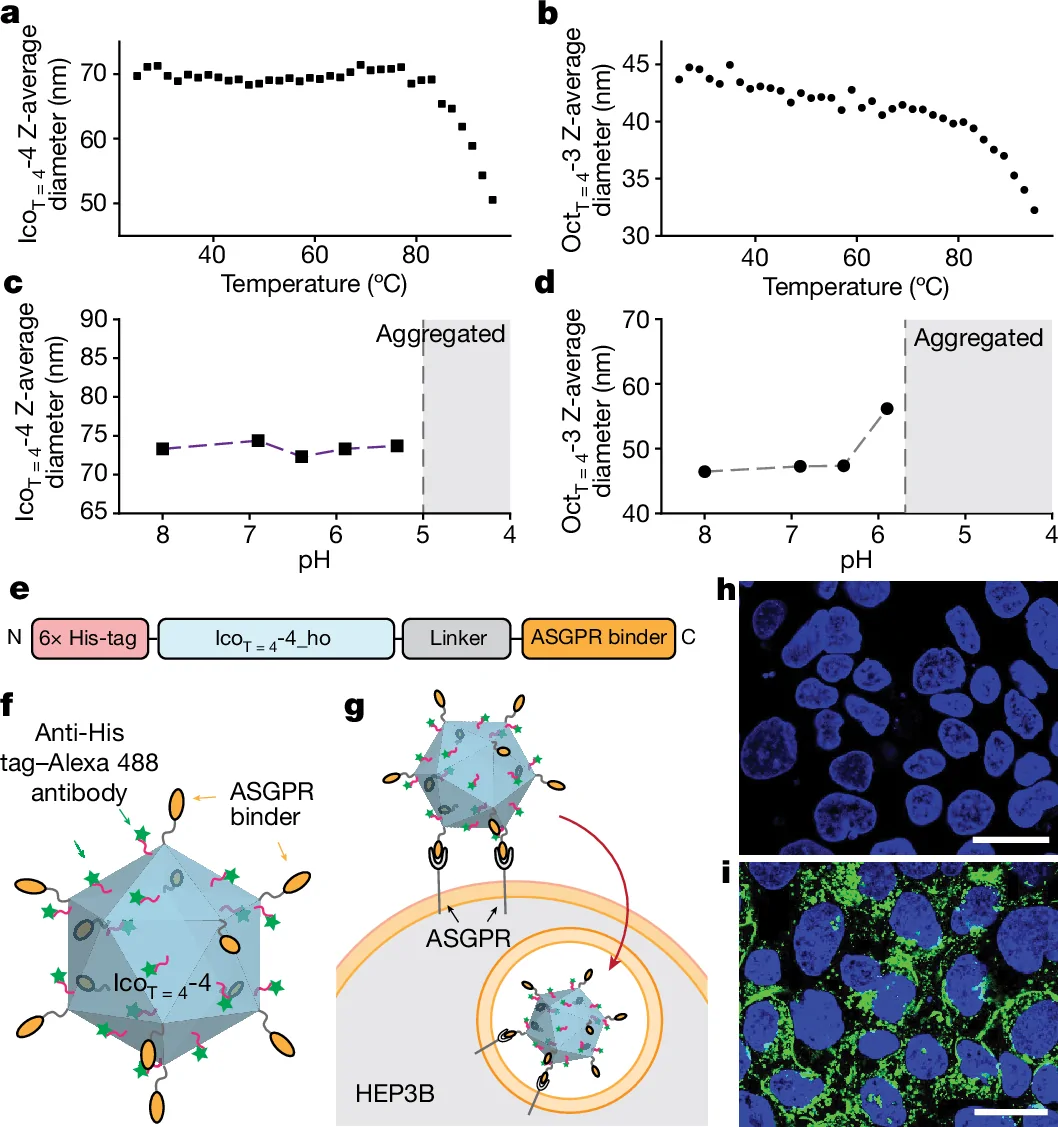
Thermal stability and pH tolerance of cages and internalization of the T = 4 icosahedral cage. **a**–**d**, Z-average diameter of Ico_T = 4_-4 and Oct_T = 4_-3 at varying temperature (**a**,**b**) and pH (**c**,**d**), measured by DLS. **e**, An ASGPR binding cage was generated by fusing the ch_ho chain of Ico_T = 4_-4 to an ASGPR-binding protein. **f**,**g**, Schematic of the ASGPR cage (**f**) and internalization of the cage in HEP3B cells (**g**). **h**,**i**, Confocal microscope images of HEP3B cells treated without (**h**) and with (**i**) the cages. Nuclei were stained with DAPI (blue), and nanocages with anti-His–Alexa-488 are shown in green. Scale bars, 30 µm (**h**,**i**).

We tested the thermal stability and pH tolerance of the Oct_T = 4_-3 and Ico_T = 4_-4 cages using DLS (Supplementary information 4.5). The Z-average diameter (*d*_Z_) of the two cages was consistent with the expected diameters of the intact cages (approximately 80 nm for Ico_T = 4_-4 and approximately 50 nm for Oct_T = 4_-3) at 25 °C (the smaller T = 1 contaminants observed by electron microscopy were not resolved by DLS probably due to the screening effect of the larger of two similarly sized particles^25^), was unchanged from 25–70 °C and then decreased above 75 °C (Fig. 6a,b). To test pH tolerance of the cages, we measured *d*_Z_ while decreasing the pH of the buffer. The Ico_T = 4_-4 *d*_Z_ of around 80 nm was unchanged from pH 8.0 to pH 5.3, but increased at pH 4.7, probably due to aggregation (Fig. 6c), whereas the Oct_T = 4_-3 of approximately 50 nm was unchanged between pH 8.0 and pH 6.4, but increased at pH 5.9 or lower (Fig. 6d). Thus, the cages are quite thermostable (melting temperatures higher than 70 °C), which should facilitate their development as delivery vehicles, and undergo pH-dependent transitions at the pH sampled in the endocytic pathway, which may be exploitable for endosomal escape.

As a first step towards exploring the use of the cages for delivery applications, we evaluated internalization by fusing one of the four components of Ico_T = 4_-4 (ch_ho) to a designed binder^26^ of asialoglycoprotein receptor (ASGPR) (Fig. 6e and Extended Data Table 6). ASGPR is an endocytic internalizing receptor that is exclusively expressed on the surface of liver cells. This receptor has been utilized for delivery of therapeutic oligonucleotides such as small interfering RNA^27^ and liver-specific LYTAC-mediated protein degradation^26,28^. Sixty ASGPR mini-binders were displayed on the outer surface of the Ico_T = 4_-4 cage through genetic fusion (Fig. 6f). We labelled the ASGPR cages with anti-His tag–Alexa-488 antibody (the subunits each have His tags) and incubated them with the hepatocellular carcinoma cell line HEP3B. By confocal imaging, we observed a substantial intracellular accumulation of the cage in a punctate localization pattern, probably indicating vesicle localization, whereas treatment with only anti-His–Alexa-488 antibody without the cage resulted in no intracellular signal (Fig. 6h,i). This result suggests that these large designed T = 4 cages can be internalized by cells for delivery or LYTAC applications.

## Conclusion

Our T = 4 assemblies are a considerable advance in compositional and structural complexity over previous nanocage designs, which have one or two distinct components and up to three distinct interfaces; the designs described here have four distinct core structural components and six distinct interfaces (Supplementary Fig. 33). Our design approach is not limited to T = 4 cages but can be extended to higher T; for example, T = 7 cages with 420 subunits can be constructed by incorporating a second heterotrimer, based on either another BGL homodimer or using a different set of designed heterotrimers^29^ (see Supplementary Figs. 49 and 50 for routes to T = 7 and T = 9). Our design approach can be applied to any set of protein oligomers that can be pseudosymmetrized and have dockable interfaces for the target point group symmetry. To our knowledge, pseudosymmetric tetrahedral and octahedral assemblies have not been observed in nature; with four distinct chains and eight termini available for functionalization, these provide starting points for sophisticated multicomponent materials. With a 75-nm diameter, our T = 4 icosahedral design has considerably more interior volume available for packaging nucleic acid and other cargoes than previously designed cages; the accompanying paper by Dowling et al.^30^ (see ref. ^31^ for a high-level comparison of approaches) describes a route to even larger, albeit less homogeneous, structures using two-component heterotrimers. For vaccine and delivery applications, the four distinct structural components of our T = 4 assemblies provide many more opportunities for further functional elaboration than the one-component and two-component nanoparticles designed to date; for vaccines, presentation of multiple antigens and immunomodulators; and for targeted delivery, incorporation of distinct designed modules for targeting and internalization.

## Methods

### Computational design

All computational design methods are described in Supplementary information 1–3.

### Protein expression

Plasmids (100 ng) were transformed into a chemically competent *E. coli* expression strain, BL21(DE3) or BL21(DE3)Star, for protein expression following the manufacturer’s protocol, with the exception of using 10 µl competent cells per reaction. Following transformation and recovery, the entire transformation products were used to inoculate 1 ml LB medium containing 100 µg ml^−1^ kanamycin and grown at 37 °C with shaking at 225 rpm overnight. Of overnight cultures, 500 µl was diluted into 50 ml TBM-5052 supplemented with 100 µg ml^−1^ kanamycin in 250 ml baffled flasks and incubated at 37 °C with shaking at 225 rpm for 18–24 h.

For systems using hetero-oligomers (pseudosymmetric hetero-oligomers, crowns and T = 4 cages), we used two different protocols: protocol 1 and protocol 2.

#### Protocol 1

Each component was transformed and incubated independently. After the incubation, we first checked relative expression level between components using SDS–PAGE (Supplementary information 4.5) based on the intensity of bands. Then, the incubated *E. coli* cultures of each component were mixed with a correct stoichiometry for target assemblies (1:1:1 for heterotrimers and crowns; 1:1:1:1 for T = 4 cages). After mixing the *E. coli* cultures, we harvested proteins following Supplementary information 4.4.

#### Protocol 2

Plasmids of all components were transformed into *E. coli* together. For example, in heterotrimer expression (three components: A, B and C), 300 ng of plasmids (100 ng of A + 100 ng of B + 100 ng of C) were transformed into 10 µl *E. coli* cells and incubated as described in this section. Then, proteins were harvested following Supplementary information 4.4.

### Protein purification

#### Immobilized metal affinity chromatography

Cultures were harvested by centrifugation at 4,000*g* for 10 min, culture supernatant was decanted, and pellets were resuspended to 30 ml in lysis buffer. Of PMSF, 300 µl (100 mM in 100% ethanol) was added immediately before sonication at 70% power for 5 min. ‘Lysate’ fractions were saved, and then lysates were clarified by ultracentrifugation at 18,000*g* at 12 °C for at least 30 min and applied to 1.5-ml Ni-NTA resin (Qiagen) pre-equilibrated with lysis buffer and packed into Econo-Pac columns (Bio-Rad) for gravity chromatography. The columns were washed twice with 15 ml wash buffer and eluted with 10 ml elution buffer. Hetero-oligomers were purified according to a similar procedure, except they used the ‘Het’ variants of the lysis, wash and elution buffers, as this was found to improve the yield of single-His-tagged complexes over nonspecific multiple-His-tagged complexes, which can arise at high concentrations and probably dominate binding to the Ni-NTA resin. Samples prepared for crystallization were treated similarly, except 500-ml cultures were used and lysate was divided among six gravity columns.

#### SEC

Immobilized metal affinity chromatography (IMAC)-eluted samples were concentrated using 10k MWCO spin concentrators and were purified using a Superdex 200 10/300 increase (for oligomers) or Superdex 6 10/300 increase (for crowns and cages) columns (Cytiva) in SEC buffer using an ÄKTA pure system (Cytiva). SEC traces were also used to qualitatively determine homogeneity and quantitatively measure total yield by A280 absorbance integrated over the collected fractions using Unicorn (Cytiva).

Note that, for T = 4 icosahedral cages (Ico_T = 4_-4), we kept the IMAC-eluted sample at room temperature for 24 h before concentrating and running SEC. The 24-h equilibration was important to generate homogeneous samples, and we think that an even longer equilibration time could be helpful to further increase the level of homogeneity. Given the complexity of this sample (4 components, 6 different interfaces and 240 subunits), a relatively slow assembly kinetics seems reasonable; thus, giving an enough equilibration time (more than 24 h) is critical to get homogeneous samples.

For all other systems, the equilibration time is not a critical issue, so we concentrated the elution sample right after IMAC and ran SEC within a few hours. However, giving enough equilibration time (more than 24 h) may not harm the sample quality, but rather possibly increase the homogeneity.

#### TEVp cleavage

Purification and mass tags were buffer exchanged into TEV buffer and cleaved with TEVp at a ratio of 1 mg TEV per 100 mg substrate for 24–72 h at room temperature. After TEV cleavage, samples were exchanged into lysis buffer and passed over a Ni-NTA gravity column and washed with 10 ml lysis buffer. Flowthrough was collected, concentrated using 10k MWCO spin concentrators and purified once again by SEC.

### Sample analysis

#### SDS–PAGE

Samples were diluted 1:1 with 2× Laemmli sample buffer (Bio-Rad) without β-mercaptoethanol, and 15 µl was loaded onto AnykD Criterion TGX Precast Midi Protein Gels (Bio-Rad). The ladder was 10 µl of Precision Plus Protein Kaleidoscope Prestained Protein Standards (Bio-Rad). Gels were run at 300 V for 18 min, then stained using the eStain L1 Protein Staining System (Genscript). Stained gels were imaged using Chemidoc XRS+ (Bio-Rad).

#### Liquid chromatography–mass spectrometry

To identify the molecular mass of each protein and thus verify sample identity and integrity, intact mass spectra were obtained via reverse-phase light chromatography–mass spectrometry on an Agilent G6230B TOF on an AdvanceBio RP-Desalting column, and subsequently deconvoluted by way of Bioconfirm using a total entropy algorithm.

#### nMS

The oligomeric state of SEC-purified and light chromatography–mass spectrometry-verified samples was analysed by on-line buffer exchange mass spectrometry in 200 mM ammonium acetate using a Vanquish ultra-high performance light chromatography system coupled to a Q Exactive ultra-high mass range Orbitrap mass spectrometer (Thermo Fisher Scientific). A self-packed buffer-exchange column was used (P6 polyacrylamide gel; Bio-Rad). The recorded mass spectra were deconvolved with UniDec version 4.2+.

#### nsEM

SEC-purified samples were diluted (0.005 mg ml^−1^ for oligomers and 0.05–0.1 mg ml^−1^ for crowns and cages) using SEC buffer immediately before application for 45 s to glow-discharged thick carbon film-coated 400 mesh copper grids (CF400-CU TH, Electron Microscopy Sciences). Grids were then stained and dried immediately twice using 2% uranyl formate. Dried grids were screened on a 120 kV Talos L120C transmission electron microscope. The E. Pluribus Unum (EPU; FEI Thermo Scientific) software was used for automated data collection. Data processing was carried out in CryoSPARC (Structura Biotechnology Inc).

#### Cryo-EM sample preparation

To prepare cryo-EM sample grids, 3 μl of 0.5–1.0 mg ml^−1^ of protein in 25 mM Tris and 300 mM NaCl at pH 8.0 was applied to glow-discharged Quantifoil R 2/2 300 mesh copper grids overlaid with a thin layer of carbon. Vitrification was performed on a Mark IV Vitrobot with a wait time of either 5 s or 7.5 s, a blot time of 0.5 s and a blot force of either 0 or −1 before being immediately plunged frozen into liquid ethane. The sample grids were clipped following standard protocols before being loaded into the microscope for imaging.

Note that we were not able to conduct cryo-EM for T = 4 tetrahedral cages, because the cages were aggregated when they are concentrated higher than 0.3 mg ml^−1^. They were stable below 0.15 mg ml^−1^, which was too dilute for conducting cryo-EM.

#### Cryo-EM data collection

Data collection was performed automatically using either Leginon^32^ or SerialEM to control either a Thermo Fisher Titan Krios 300 kV TEM equipped with a standalone K3 Summit direct electron detector with an energy filter or a Thermo Fisher Glacios 200 kV equipped with a standalone K3 Summit direct electron detector^33^. Both samples were collected using counting mode, with random defocus ranges spanning between −0.7 µm and −1.8 μm using image shift, with one shot per hole on the Glacios for Oct_T = 4_-3 or multiple shots per hole on the Titan Krios for Ico_T = 4_-4. Movies (*n* = 1,320 and 1,048) were collected with a pixel size of 0.44 Å for Oct_T = 4_-3 with a total dose of approximately 50 e^−^ Å^−2^, and 6,678 movies were collected with a pixel size of 0.84 Å for Ico_T = 4_-4 with a total dose of approximately 61 e^−^ Å^−2^.

### Reporting summary

Further information on research design is available in the Nature Portfolio Reporting Summary linked to this article.

## Online content

Any methods, additional references, Nature Portfolio reporting summaries, source data, extended data, supplementary information, acknowledgements, peer review information; details of author contributions and competing interests; and statements of data and code availability are available at https://doi.org/10.1038/s41586-024-07814-1.

## Supplementary information

Supplementary Information This file contains Sections 1–4; Supplementary Figs. S1–S51; Supplementary Table S1–S13 and Supplementary References

Reporting Summary

Supplementary Fig. 1

## Figures and Tables

**Extended Data Table 1:**
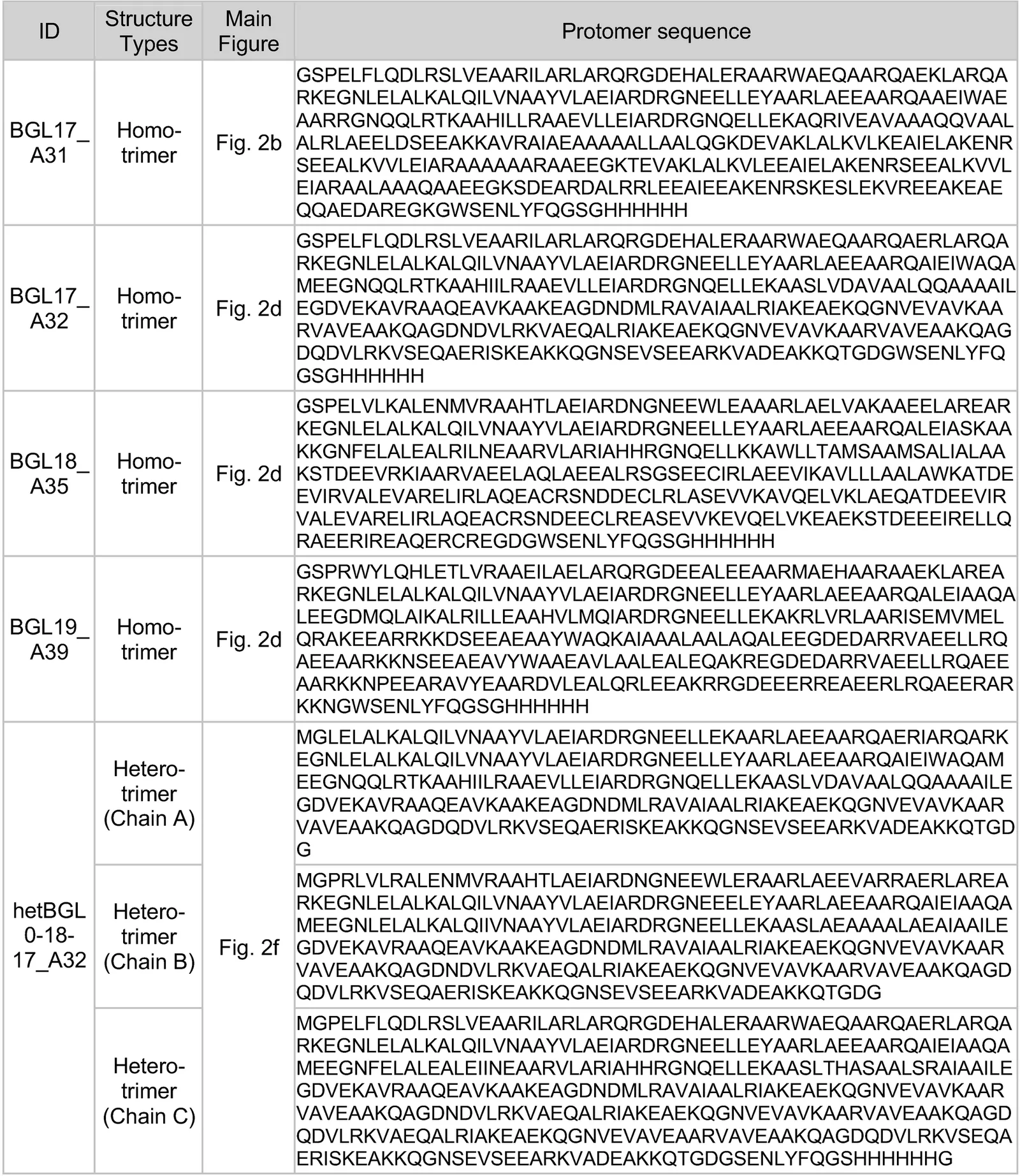
Designed protein sequence

Designed sequences of C3 homotrimers and pseudo-symmetric heterotrimer shown in the main figures.

**Extended Data Table 2:**
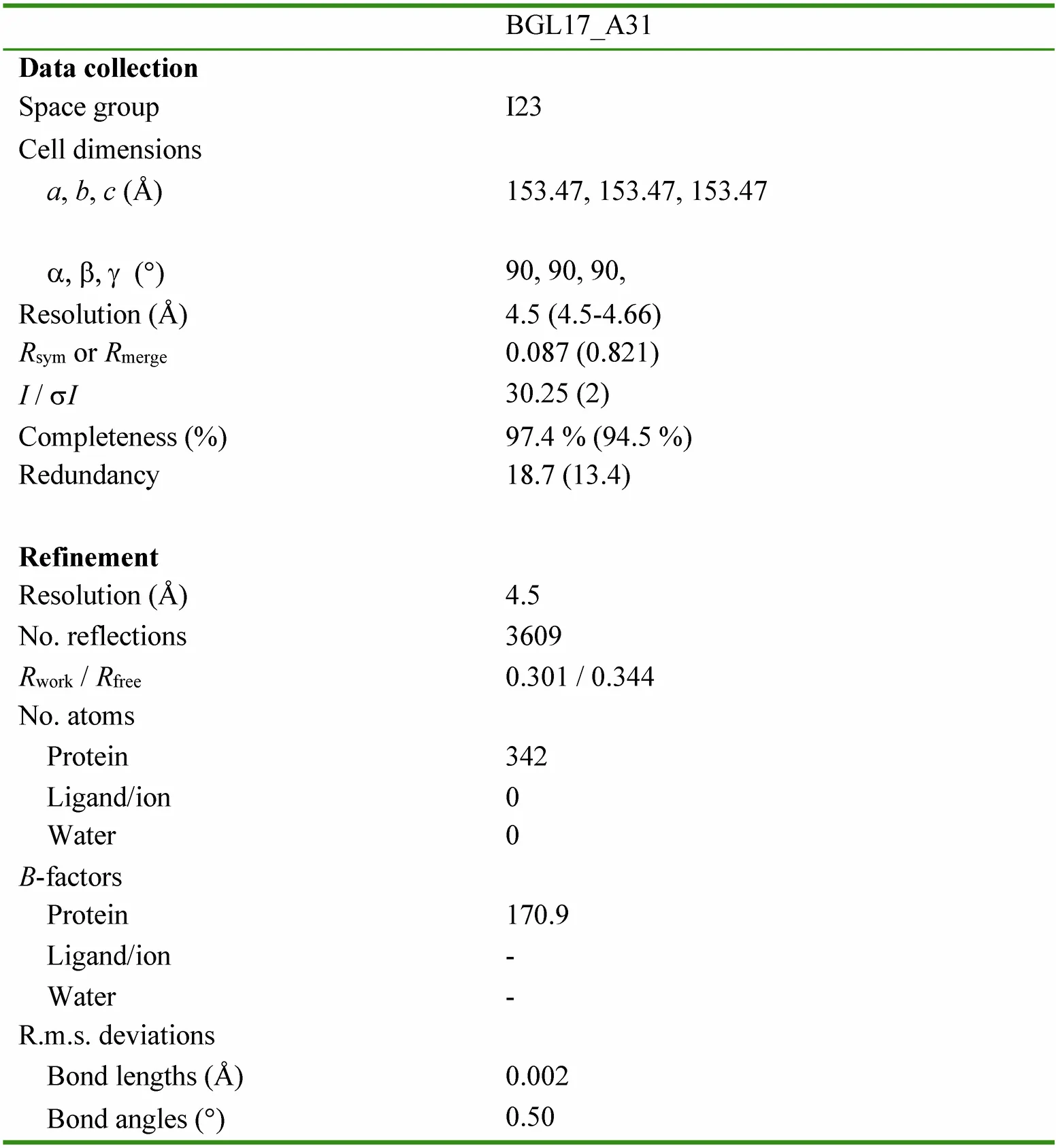
Crystallographic data

BGL17_A31 (PDB ID: 8FLX).

**Extended Data Table 3:**
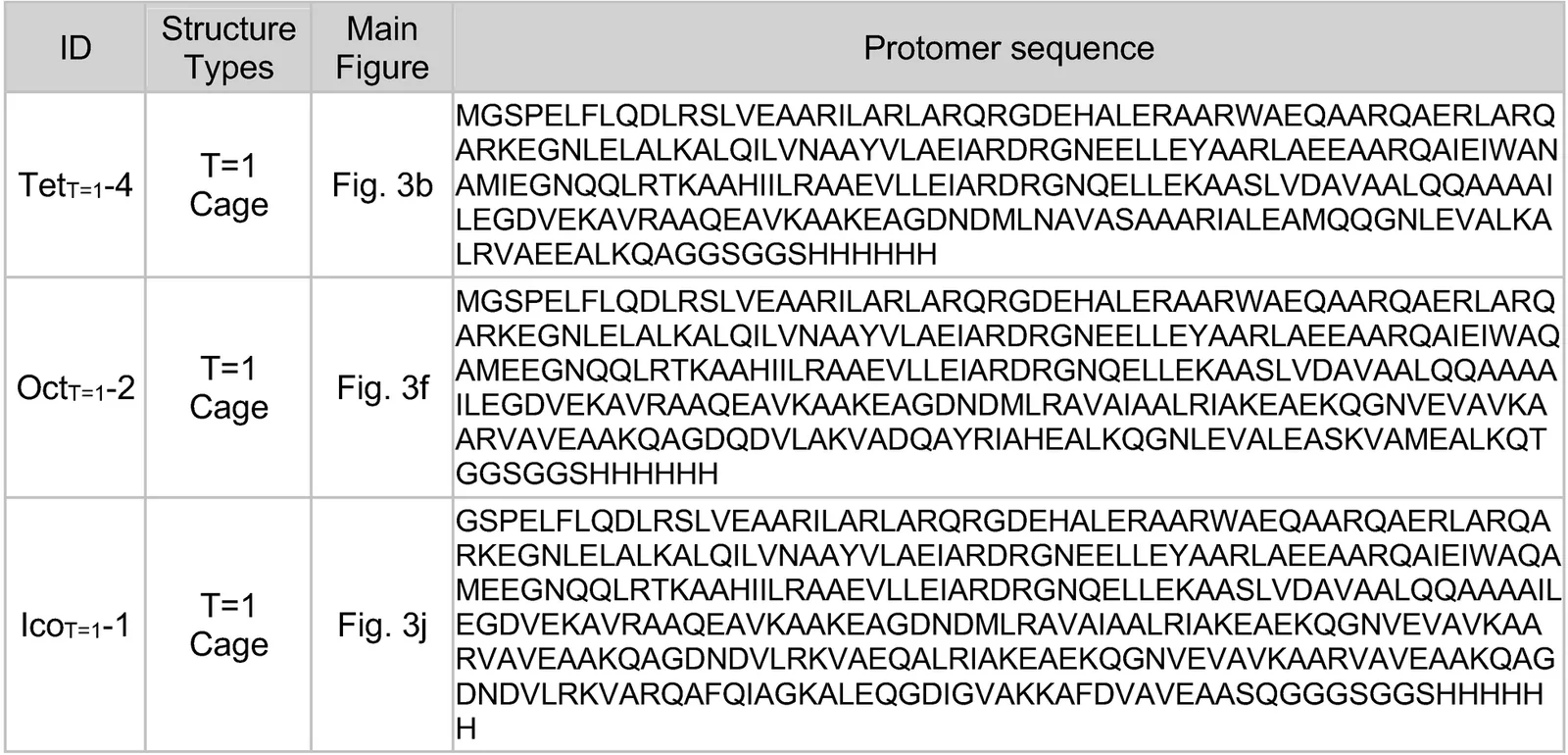
Designed protein sequence

T = 1 cages shown in the main figures.

**Extended Data Table 4:**
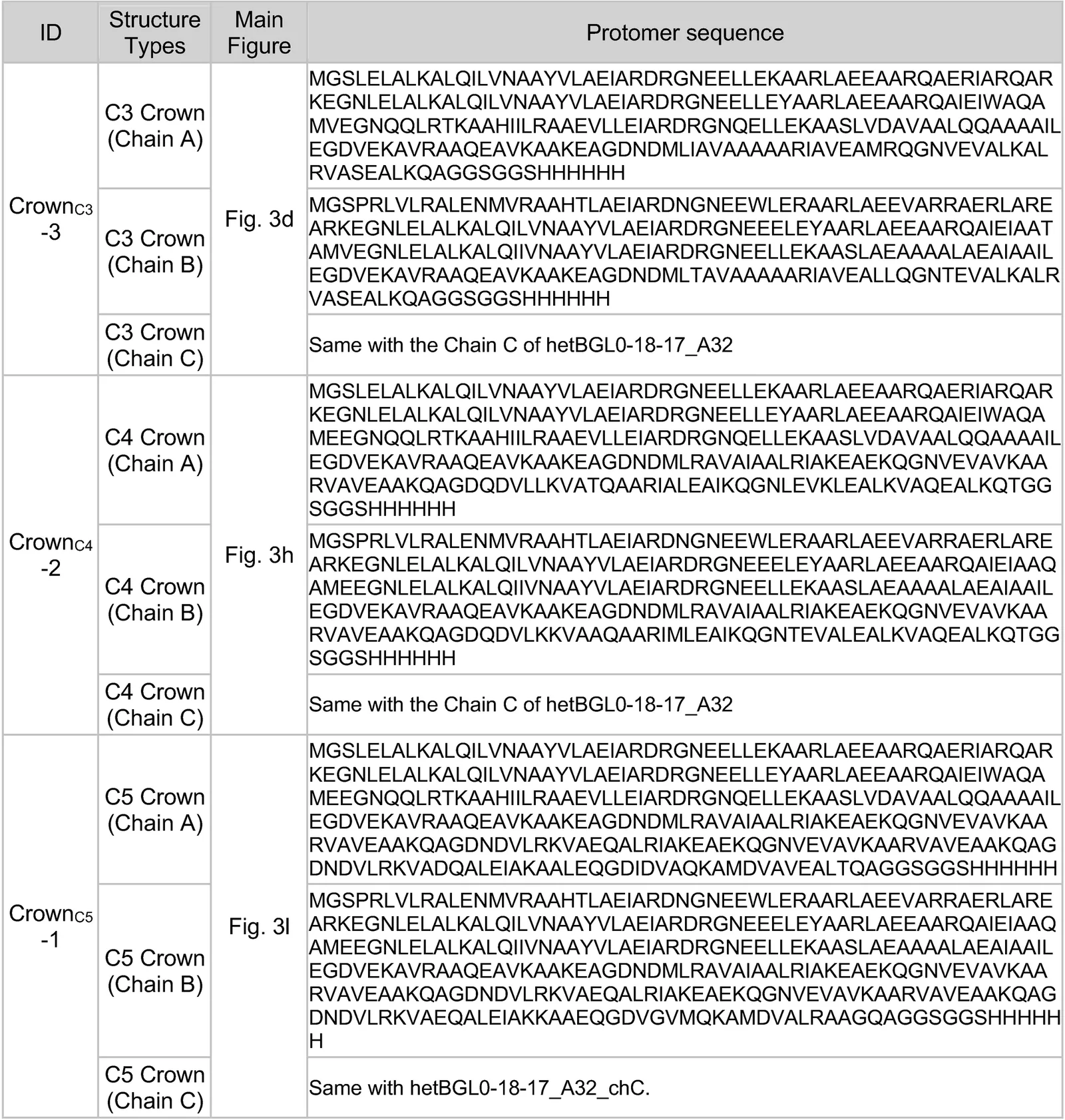
Designed protein sequence

Crowns shown in the main figures.

**Extended Data Table 5:**
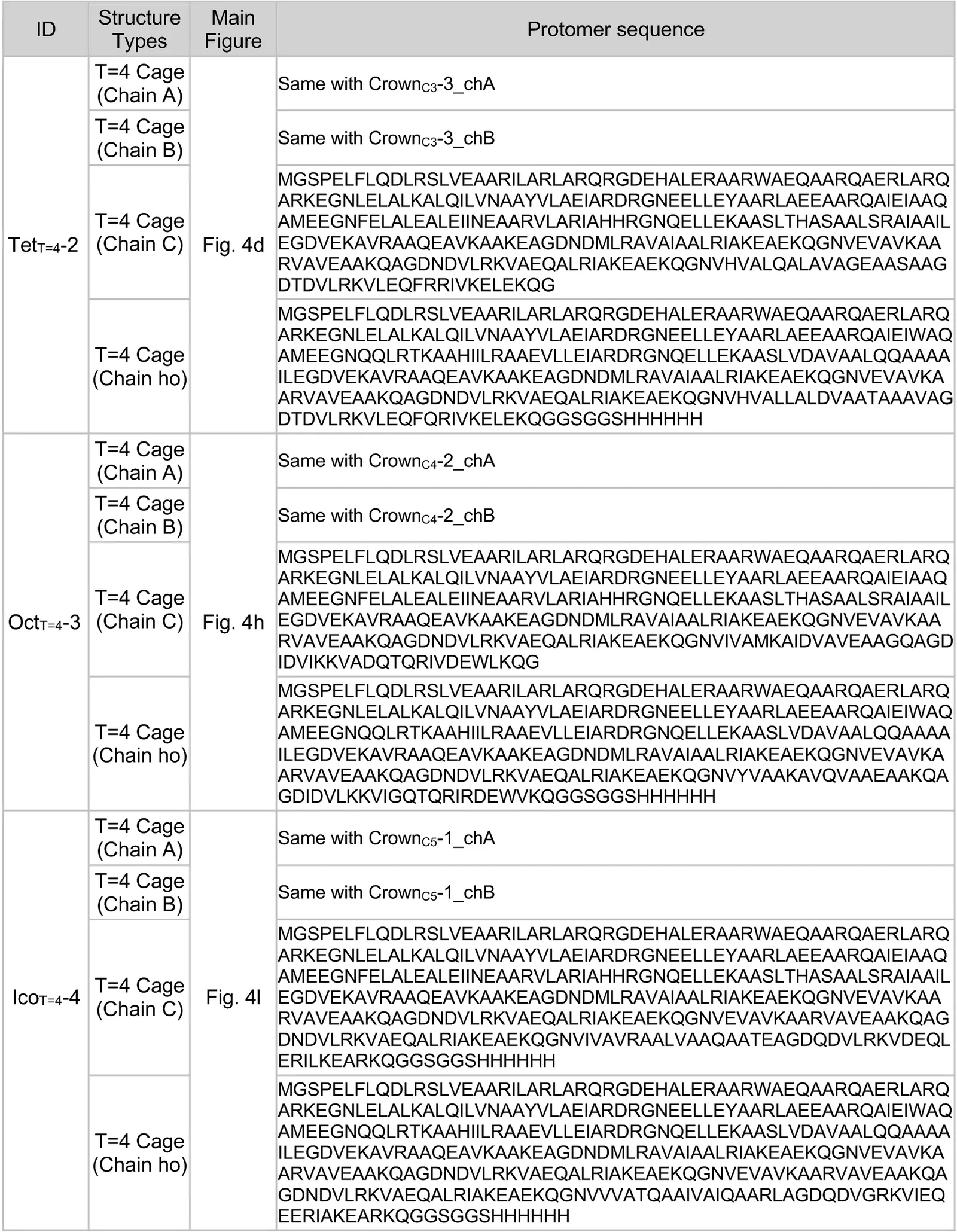
Designed protein sequence

T = 4 cages shown in the main figures.

**Extended Data Table 6:**
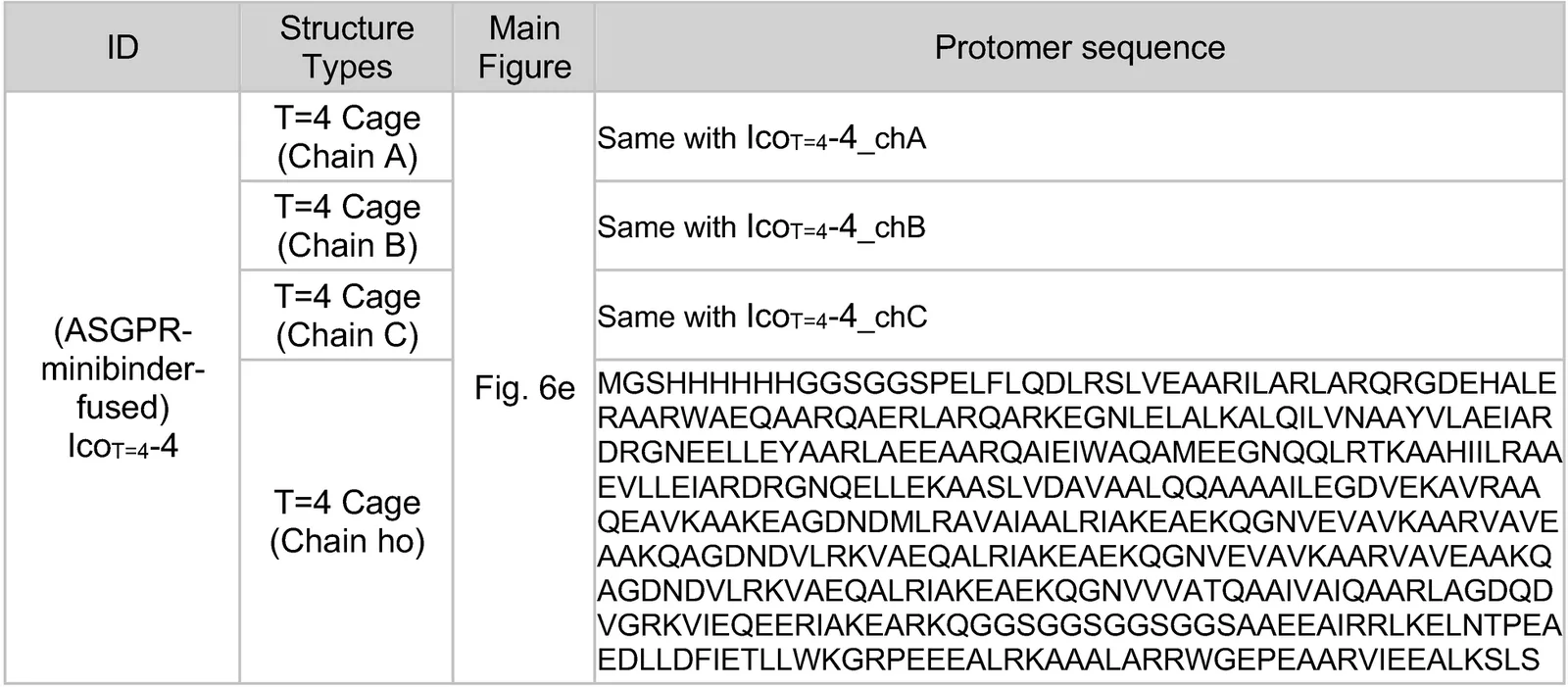
Designed protein sequence

The ASGPR-cage in which one of the four components (chain_ho) of Ico_T=4_-4 is fused to an ASGPR minibinder.

**Extended Data Table 7:**
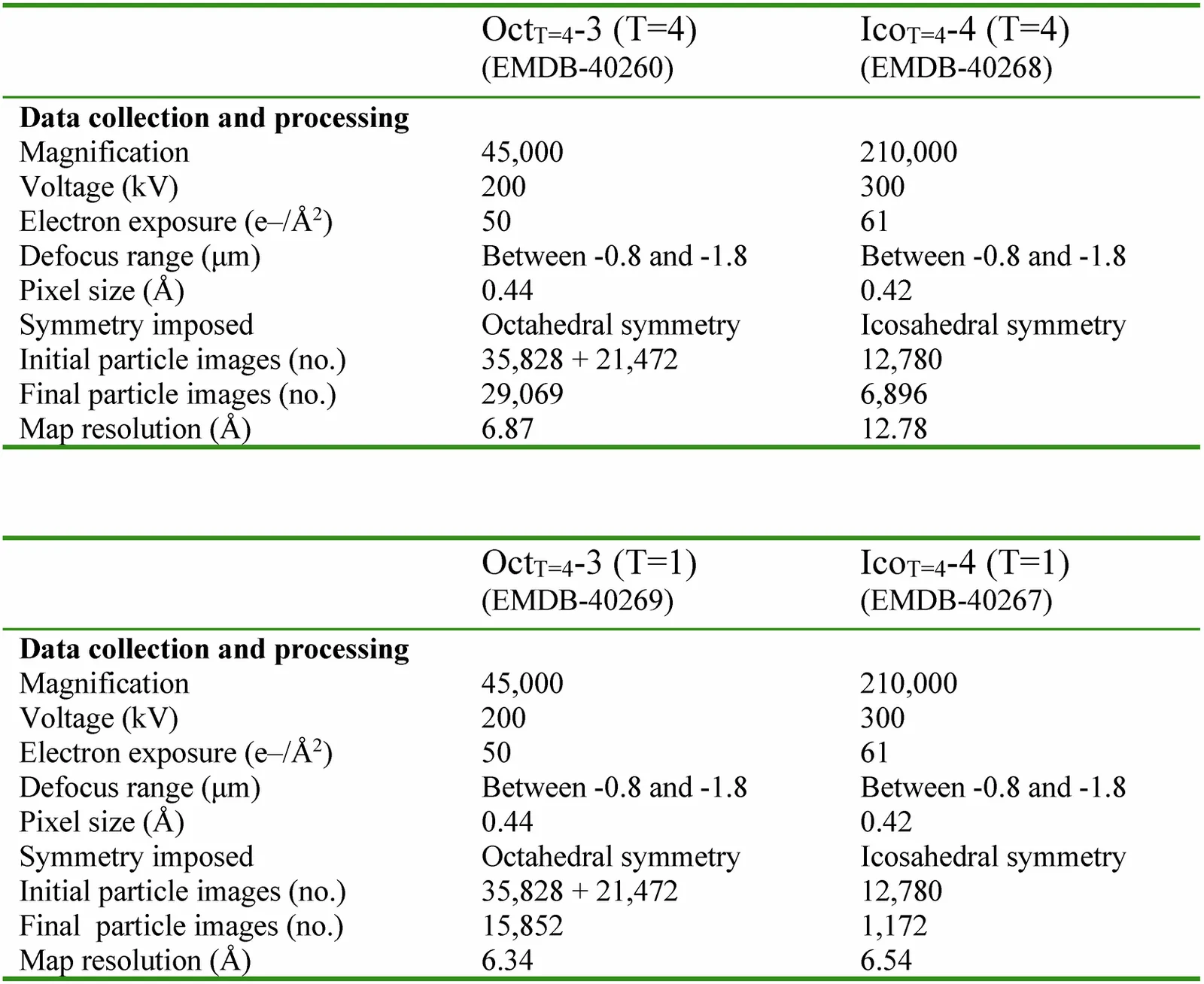
CryoEM data collection information

T = 4 cages (Oct_T=4_-3 and Ico_T=4_-4) and off target small cages (T = 1 cages) found in Oct_T=4_-3 and Ico_T=4_-4 systems.

## Data Availability

Crystallographic data of BGL17_A31 have been deposited in the Protein Data Bank (ID 8FLX). Electron microscopy maps have been deposited in the Electron Microscopy Data Bank (accession codes EMD-40260, EMD-40267, EMD-40268 and EMD-40269).

## Code availability

The source code from RPXdock for the protein docking is freely available at https://github.com/willsheffler/rpxdock. The source code from ProteinMPNN for the sequence design is freely available at https://github.com/dauparas/ProteinMPNN.
