# Supplementary material for: Four-component protein nanocages designed by programmed symmetry breaking
Source: Nature. 2024 Dec 18;638(8050):546–52. doi: 10.1038/s41586-024-07814-1 (PMC11821509; doi:10.1038/s41586-024-07814-1)
Supplement: Supplementary file 3 [file 41586_2024_7814_MOESM3_ESM.pdf]

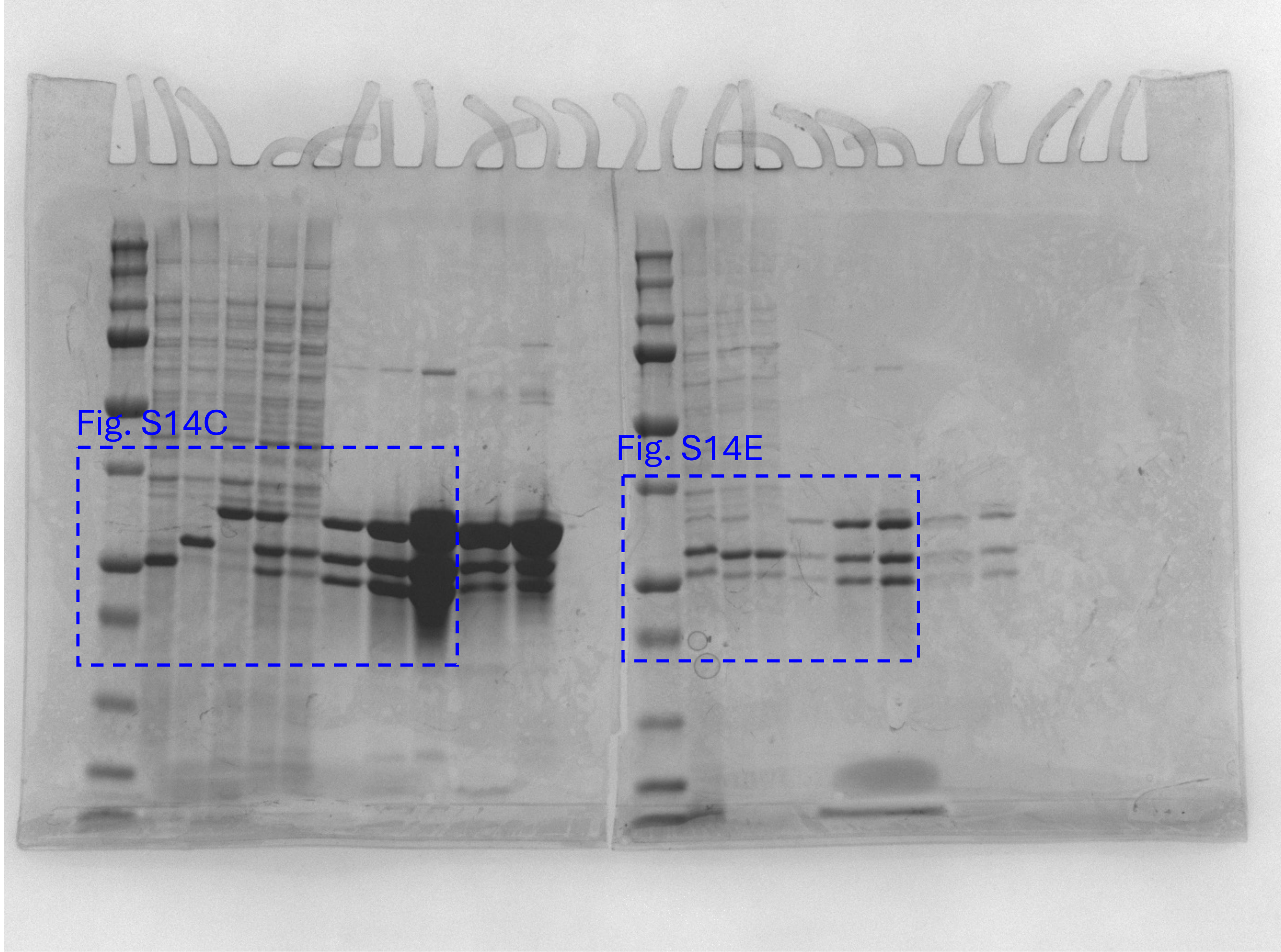
Fig. S14C
Fig. S14E

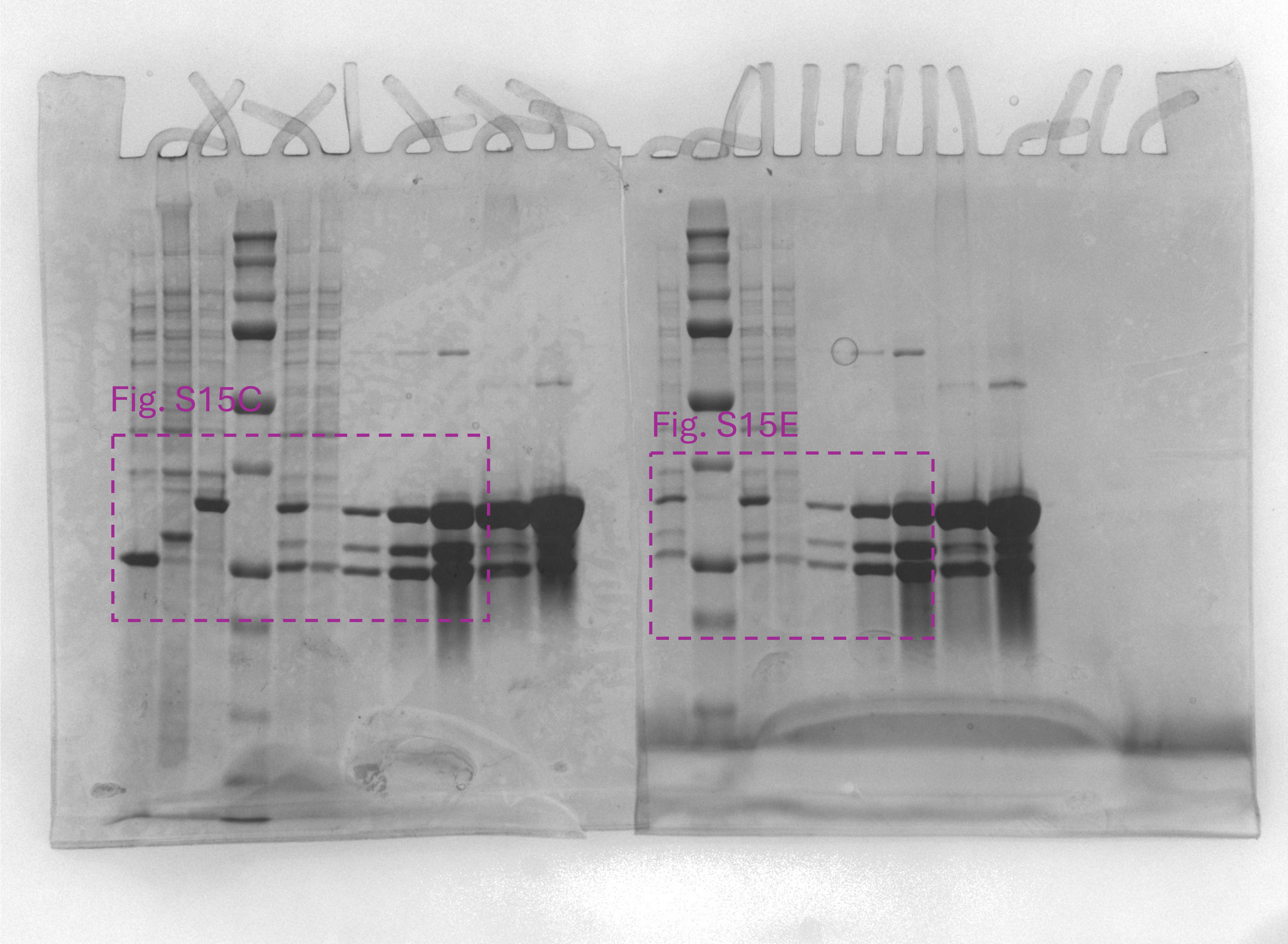

Fig. S15C
Fig. S15E

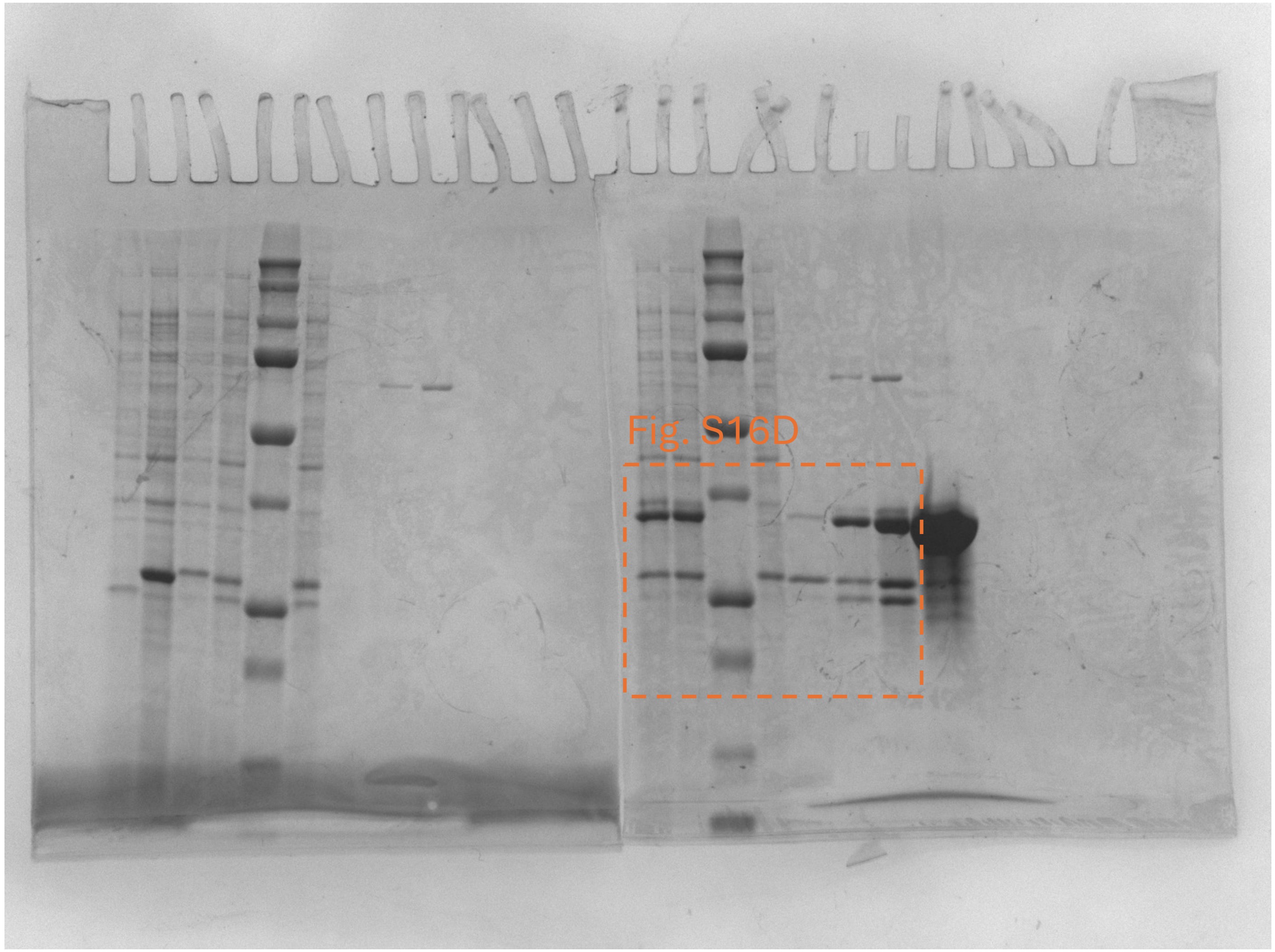
Fig. S16D

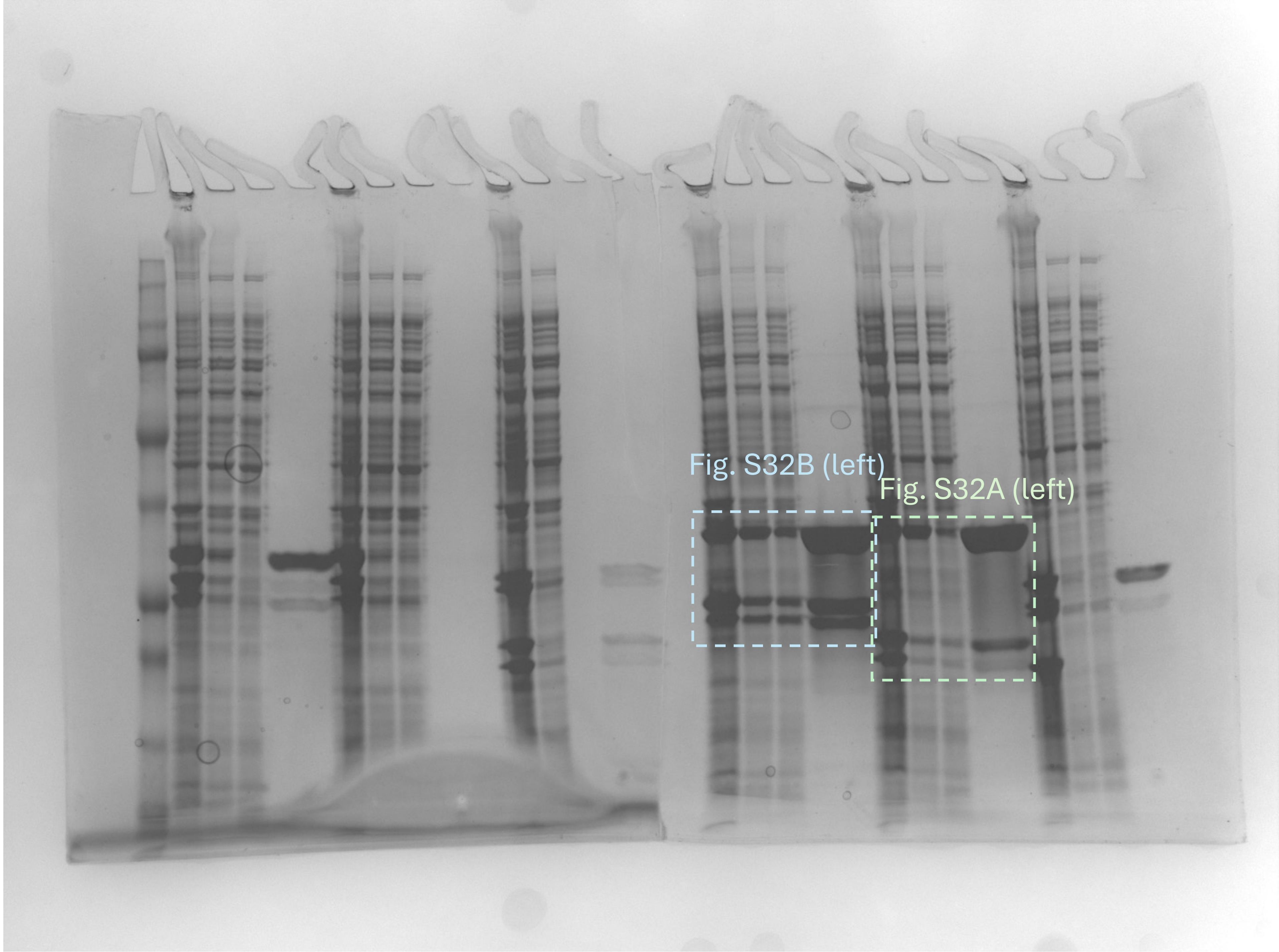

Fig. S32B (left)
Fig. S32A (left)

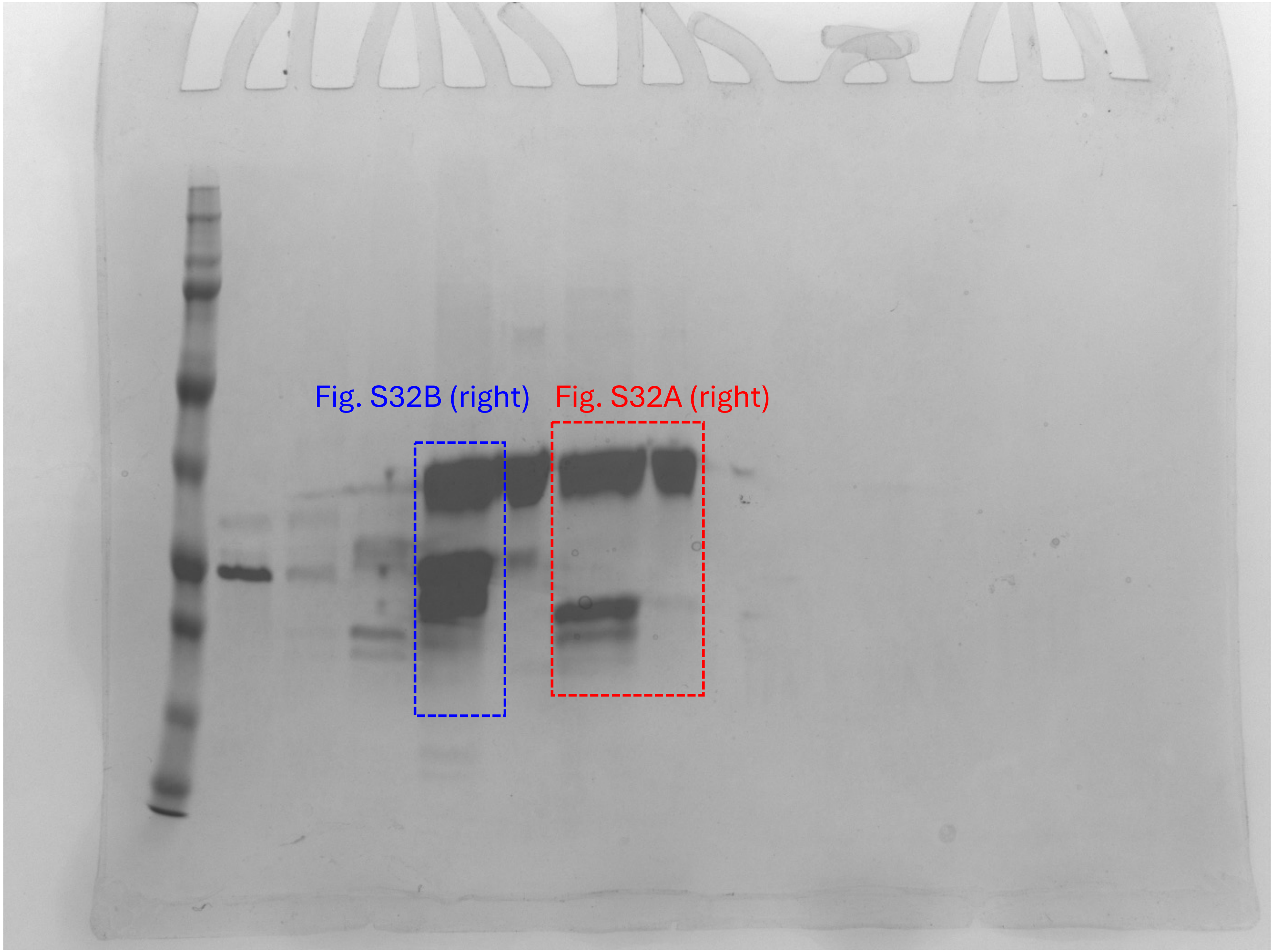
Fig. S32B (right)
Fig. S32A (right)

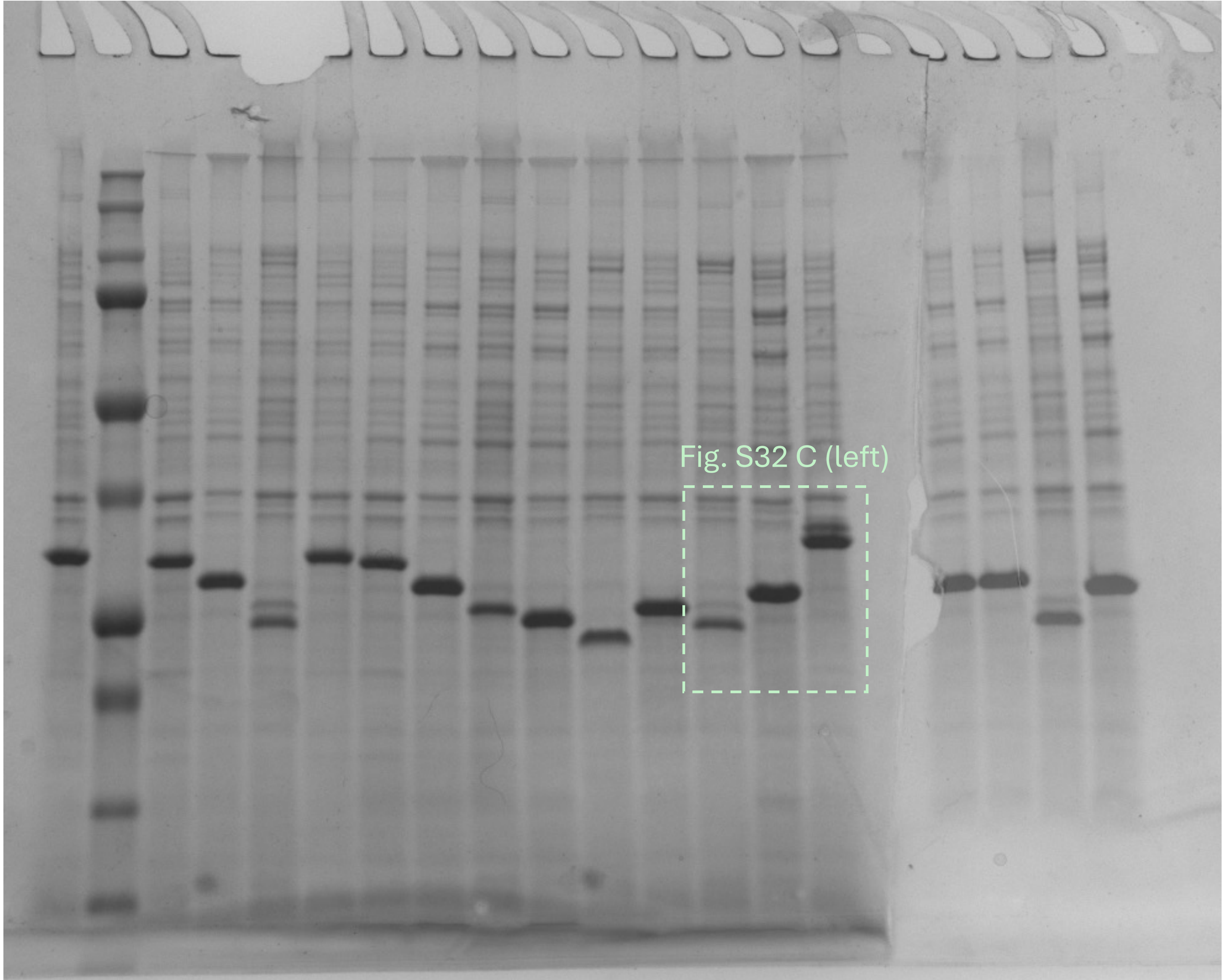
Fig. S32 C (left)

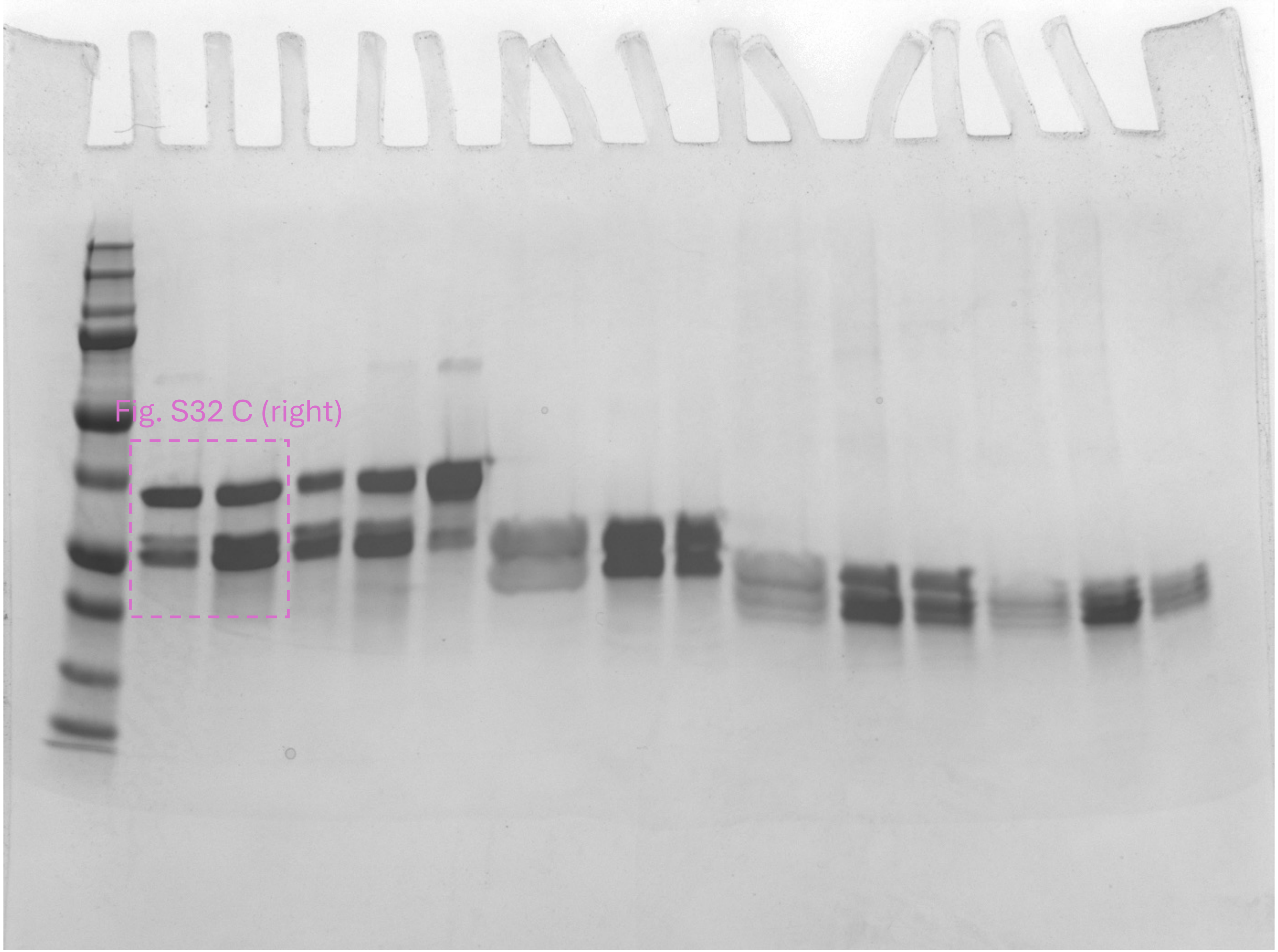
Fig. S32 C (right)

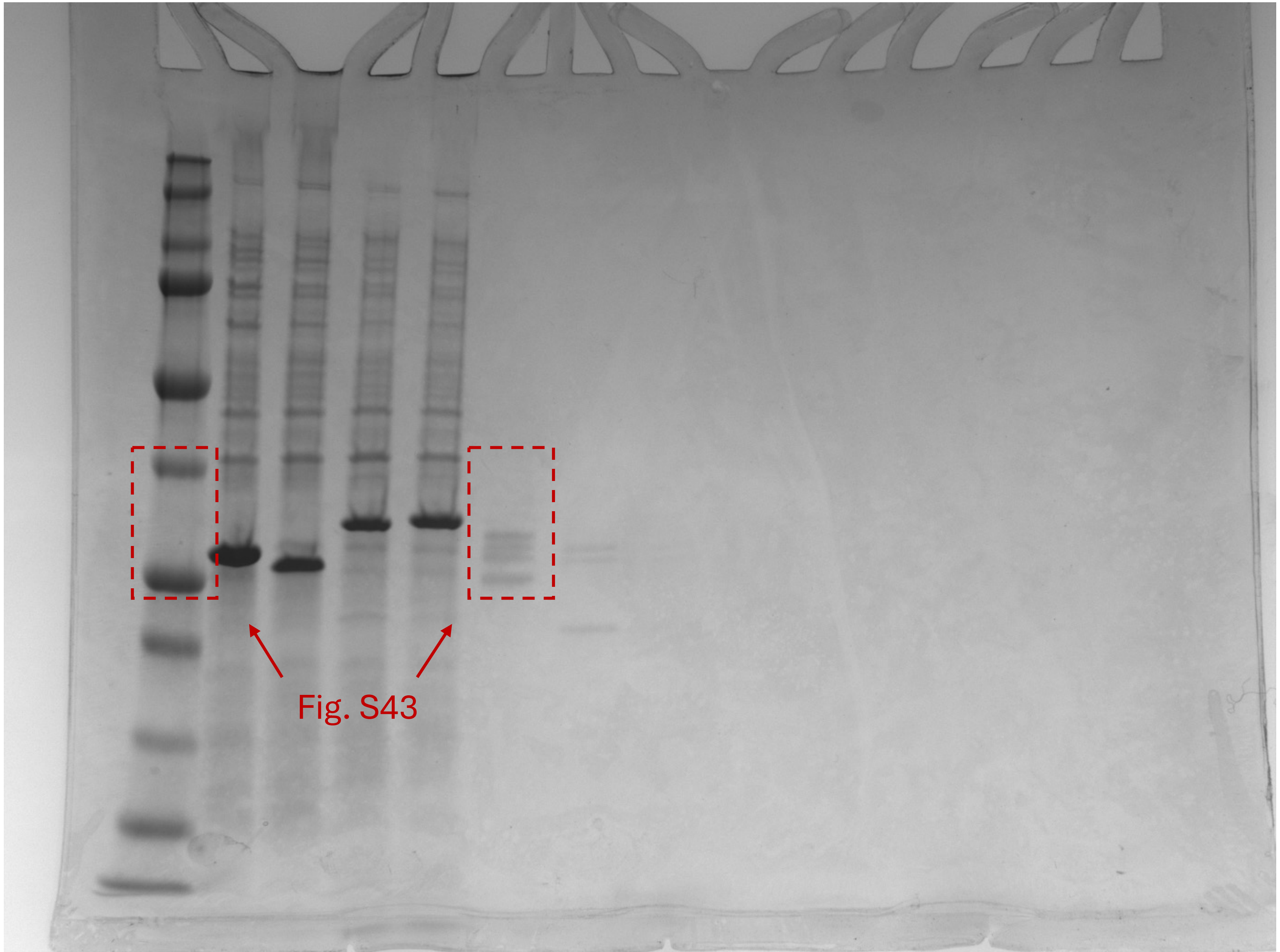
Fig. S43
